# Ecological and Genomic Evidence of Cryptic Speciation in the Potato Psyllid (*Bactericera cockerelli*)

**DOI:** 10.1111/eva.70334

**Published:** 2026-10-20

**Authors:** Jaimie R. Kenney, Giulia Scarparo, Marco Molfini, Mona Tran, Steven Tran, Simoné Murguia, Marco Gebiola, Poulami Sarkar, Jessica Purcell, Kerry E. Mauck

**Affiliations:** ^1^ Department of Entomology University of California, Riverside Riverside California USA; ^2^ Department of Biological Sciences Diablo Valley College Pleasant Hill California USA; ^3^ Institute of Organismic and Molecular Evolution Johannes Gutenberg University Mainz Germany; ^4^ Department of Microbiology and Plant Pathology University of California, Riverside Riverside California USA; ^5^ Department of Agricultural Sciences University of Naples Federico II Portici NA Italy

**Keywords:** ddRADseq, gene flow, haplotype, reproductive isolation, zebra chip disease

## Abstract

The potato psyllid (*Bactericera cockerelli*) is a crop pest that serves as a vector for the bacterial pathogen *Candidatus* Liberibacter solanacearum (CLso), which causes disease in solanaceous and apiaceous crops. This psyllid species carries substantial haplotypic diversity based on mitochondrial cytochrome oxidase 1 (mtCOI) sequences, associated with strong geographic and temporal separation. Using mtCOI sequencing and genomic ddRADseq analysis of 111,587 SNPs across 590 individuals collected from native Solanaceae and cultivated crops at 50 geographically paired sites in California, we show that the previously described haplotype diversity in *B. cockerelli* may represent at least three genetically distinct cryptic species. These three lineages exhibited extremely high genomic *F*_ST_ values across all 13 chromosomes and are strongly partitioned by the host plant. The Northwestern lineage is strictly associated with the native perennial *Solanum umbelliferum* , while the Western lineage is associated with solanaceous crops, and the Southwestern lineage moves between native and cultivated hosts in desert regions. Hybridization is also rare, suggesting reproductive isolation. SNP and divergence analyses for the obligate bacterial symbiont support conclusions of the host nuclear and mtCOI sequence analyses. We also detected five CLso variants in psyllid samples. The Northwestern lineage housed G, Sumb2, F and B variants, while the Western lineage housed only B and A variants. The largest diversity of CLso variants occurred in coastal regions where psyllid lineages overlap. Exploring the ecology and CLso associations of this potential cryptic species complex provides a foundation for improving biosecurity and management of potato psyllids and CLso pathogens.

## 1 Introduction

Insects constitute about half of all described, extant species living on Earth (Bánki et al. 2025). Despite insects being ubiquitous and essential components of nearly every ecosystem, our knowledge of their true biodiversity is incomplete. One factor that slows progress in this area is the prevalence of cryptic insect species, which are distinct species that cannot be reliably distinguished by morphological characteristics, but may differ markedly in other less obvious features, including (but not limited to) genetics, behavior, host range and ability to transmit pathogens or parasites (Paterson et al. 2016; Wang et al. 2019; Xue et al. 2022). Lack of resolution of the extant biodiversity could limit not only our ability to preserve this biodiversity, but also undermine pest management efforts, particularly when such species represent a significant agricultural threat (Karbstein et al. 2024; Cheng et al. 2025; Hending 2025). For example, the sweet potato whitefly ( *Bemisia tabaci* ), a cosmopolitan sap‐feeding pest and vector of plant viruses, causes billions of dollars in crop losses and has a particularly severe impact on food security in the global south (Perring et al. 2018; Perier et al. 2022; Kavallieratos et al. 2024). Research over the last two decades has shown that *B. tabaci* is not just one species, but a complex of putatively up to 44 cryptic species with distinct geographic distributions, host preferences, virus associations, environmental tolerances and insecticide resistances (see (Brown et al. 2023; Wang et al. 2025) and references therein). Understanding the diversity and distribution of this species complex revealed why many control efforts against whiteflies and whitefly‐borne viruses fail.

The example of *B. tabaci* illustrates how critical it is to understand the true diversity of organisms transmitting plant pathogens in the environment. Estimating this diversity is not trivial, but attempts have been made to leverage existing datasets to predict how many cryptic species we should expect to discover, if we are willing to look. A recent meta‐analysis estimated the average number of cryptic species per morphology‐based species for major insect orders using studies that performed molecular‐based species delimitations, with results weighted to account for the relative richness of each order (Li and Wiens 2022). Using data on 25 genera across eight orders (covering 73.4% of described insect species), the authors estimated that there are, on average, 3.11 cryptic species for every morphology‐based species so far described (range of 1.52–6.25). The highest ratio of cryptic species documented for any order was 6.25, in the order Hemiptera (Li and Wiens 2022).

If these estimates are accurate, they suggest that insect vectors of plant pathogens in the order Hemiptera may be even more likely to exhibit cryptic species diversity than other taxa. Other lines of evidence support this idea. Hemiptera are primarily phytophagous and there is substantial evidence that phytophagous insects undergo rapid radiations (including sympatric speciation) through host shifts (Forbes et al. 2017). Additionally, infections in plants by Hemiptera‐transmitted pathogens are known to modify plant phenotypes by enhancing their suitability to vector hosts (e.g., by suppressing plant defenses or increasing nutrient content) (Davis et al. 2017; Mauck, Kenney, and Chesnais 2019). This could help facilitate host plant shifts and subsequent diversification of the phytophagous vector.

Despite the meta‐analysis by Li and Wiens (2022) covering ~73% of described insect species, the Hemiptera dataset included only four genera. There is also limited representation of virus‐vectoring insects, with the exception of the extremely well studied pest genus *Bemisia*. Thus, while the study by Li and Wiens (2022) and other work (Kosovac et al. 2018; Trivellone and Dietrich 2021) raise the intriguing possibility that the phytophagous vector lifestyle facilitates cryptic species diversity, we need studies on more hemipteran taxa to evaluate this prediction. This research avenue has clear applied relevance due to the impacts of insect vectors and the pathogens they transmit on food production, as well as their probable (but poorly studied) impacts on wild plant communities and conservation efforts (Vincent et al. 2014; Malmstrom and Alexander 2016; Alexander et al. 2017).

The present study addresses this knowledge gap by exploring molecular and ecological evidence of cryptic speciation in the potato psyllid, *Bactericera cockerelli* (Šulc) (Hemiptera: Triozidae), a phytophagous pest of solanaceous crops and primary vector of the bacterial plant pathogen *Candidatus* Liberibacter solanacearum (CLso) in North America (Wenninger and Rashed 2024). This species is thought to be native to western North America and was first described (as *Trioza cockerelli* in the family Psyllidae) following collections from an infestation on cultivated peppers in Colorado (Šulc 1909). By the 1920s *B. cockerelli* had emerged as a serious pest of potatoes in Utah, Colorado, and Wyoming and crop infestations expanded to include California in the 1930s and 40s (Wallis 1955). Severe outbreaks regularly led to crop failures until the late 1940s, when use of insecticides to manage *B. cockerelli* became commonplace (Wallis 1955). Losses to *B. cockerelli* remained minimal until the late 1990s and early 2000s, when crops in the central U.S., Washington, California, Baja (Mexico) and even Ontario, Canada suddenly experienced large infestations (Butler and Trumble 2012). Following this reemergence as a pest, researchers conducted genotyping studies using a 500 bp region of the mitochondrial cytochrome oxidase 1 (mtCOI) gene to understand the genetic diversity of *B. cockerelli* in the United States based on psyllid collections from crops. This resulted in identification of four psyllid haplotypes named based on the geographic regions where the samples originated (Central, Western, Northwestern and Southwestern) (Swisher, Munyaneza, and Crosslin 2013; Swisher et al. 2014).

The emergence and spread of zebra chip disease (ZCD), which is caused by CLso infection, occurred concurrently with reemergence of *B. cockerelli* as a pest in crops in the 1990's. ZCD is characterized by abnormalities in the medullary rays and vascular ring in potato tubers that result in a zebra‐stripe appearance when the tuber is sliced and fried (Munyaneza et al. 2007). The disease was first observed in Saltillo, Mexico in 1994, and rapidly expanded to Texas, then most of the potato‐producing areas of the Western U.S., in the ensuing decade. The CLso causal agent was first identified by Hansen et al. (2008), who also found evidence of transovarial transmission of CLso from female psyllids to their offspring. Later studies confirmed that ingested CLso bacterial cells can cross the gut barrier and establish as an intracellular symbiont (Sarkar and Ghanim 2020), suggesting a long evolutionary history of CLso with psyllid hosts.

Since the initial discovery of the causal agent of ZCD, 16 biologically and genetically distinct CLso variants have been discovered in association with plant and psyllid hosts around the world (Mauck et al. 2024; Tran et al. 2024). In North America and areas where the potato psyllid is introduced, ZCD is caused by the A or B variants of CLso transmitted by *B. cockerelli* (Wenninger and Rashed 2024). However, screening of herbarium specimens of native and introduced *Solanum* species collected throughout the Southwest revealed asymptomatic infections by a novel variant of CLso (G) in the native perennial, *Solanum umbelliferum* (Mauck, Sun, et al. 2019). Specimens dating back to 1970 tested positive for the G variant of CLso (34 years prior to the first observations of ZCD in crops in the U.S.) and this variant has never been reported infecting crops. The G variant, and an additional new CLso variant (Sumb2) were later detected, again as asymptomatic infections, in contemporary populations of *S. umbelliferum* growing in California, but the A and B variants that cause disease in crops were not present (Kenney et al. 2024). Kenney et al. (2024) also used mtCOI sequencing to show that potato psyllids collected from *S. umbelliferum* are of the Northwestern and Southwestern haplotypes, which are genetically distinct from the Western haplotype that had previously been found in crop habitats in California and most other Western states (Liu et al. 2006; Liu and Trumble ; Swisher et al. 2012).

These studies document distinct partitioning of CLso variants and *B. cockerelli* haplotypes between crop and wild habitats, even at local scales, leading to the hypothesis that *B. cockerelli* haplotypes in the Southwest may have diversified into a cryptic species complex. We can predict that different psyllid haplotypes may strictly associate with specific host plants, with the Western haplotype preferring crops (particularly peppers and potatoes), and the Northwestern and Southwestern haplotypes dominant in populations of wild plant hosts (primarily *S. umbelliferum* and *Lycium* spp.). These perennial hosts are suitable for *B. cockerelli* during late fall, winter and very early spring—completely off‐cycle from the production of solanaceous crops in the same geographic area (late spring, summer, early fall). Psyllids that use hosts in leaf and bloom in the winter may migrate to shelter plants (e.g., conifers) that provide moisture and some nutrients, but are not reproductive hosts, during the summer period (Burckhardt et al. 2014). This temporal separation during the mating periods could also reinforce reproductive isolation due to host plant preferences. Cryptic species partitioning by both host plant species and season may also drive differences in CLso associations and the relative risks of new CLso pathogen emergence in spatially contemporaneous crops.

To test the hypothesis that *B. cockerelli* has undergone cryptic speciation in the Southwestern U.S., we completed a large‐scale *B. cockerelli* sampling effort in California using 49 geographically paired sites containing wild Solanaceae (primarily *S. umbelliferum* ) or solanaceous crops (tomato, potato and bell pepper). Although our sampling is limited to just a small portion of the current *B. cockerelli* global distribution, the selected area is unique in having both intensive production of solanaceous specialty crops near key wild hosts that are native to the region, as well as three of the four known psyllid haplotypes (the fourth was also found in our sampling efforts). The location is therefore ideal for testing our hypothesis and may be the only location in the native range in which such a study is possible. Following sampling, we combined mtCOI sequencing with double‐digest restriction enzyme‐associated DNA sequencing (ddRADseq) to characterize psyllid population structure and infer frequency of potato psyllid movement between cultivated and wild Solanaceae populations throughout the state. Finally, to understand how potato psyllid population structure might influence patterns of CLso transmission, we screened these psyllid populations for CLso and used multi‐locus sequence typing (MLST) to determine patterns of association between potato psyllid populations and specific CLso variants.

## 2 Materials and Methods

### 2.1 Psyllid Sampling Procedures

Potato psyllid sampling locations were selected using a variety of methods. An extended description of sampling habitats and seasonality can be found in the Methods in Supporting Information. Populations of the native perennial *S. umbelliferum* were identified throughout California using iNaturalist.org. Populations near major highways and in University of California (UC) Natural Reserves and county parks were preferred, due to ease of access and logistical feasibility of obtaining permission to collect biological samples at these locations. Populations of the native desert Solanaceae *Lycium andersonii* and *Physalis crassifolia* in Southern California were also located using iNaturalist observations. Scientific research permits were obtained before sampling psyllids from native plant populations in State or National Parks. Contact information for California growers of potato, tomato and bell pepper, or their pest control advisors (PCAs), was obtained by contacting UC Extension specialists and county Farm Bureaus. In many cases the authors accompanied UC extension specialists to farms where they already had permission to sample insects for UC research. Otherwise, growers and pest control advisors were contacted directly by the authors to obtain permission to sample specific agricultural fields. Efforts were made to sample at least one native plant population and one field of solanaceous crops per county where solanaceous crops are commercially grown (all site locations shown in Figure S1 with coordinates and site information in Table S1).

At each psyllid sampling location, depending on the height and shape of the host plant, plants were either shaken over a canvas insect beating sheet (BioQuip) or swept with an insect net. Any potato psyllids observed on the beating sheet or caught in the net were collected directly into 33 mL plastic vials using a small insect aspirator (BioQuip). Vials were placed in a cooler of ice for live transport back to the lab. At the lab, psyllids were counted and photographed and placed in tubes of 95% ethanol and stored at −80°C to await DNA extraction. In rare cases (some tomato fields), we deployed yellow sticky cards for several days if beating sheet sampling efforts resulted in no collected individuals.

### 2.2 DNA Extraction

Individual psyllids were air‐dried from ethanol and then placed in 1.7 mL Eppendorf tubes and submerged in liquid nitrogen. While still in the liquid nitrogen, psyllids were manually ground for approximately 30 s using sterile polypropylene pellet pestles. After grinding, 180 μL Buffer ATL (Qiagen) and 20 μL of proteinase K (Qiagen) were added to each tube. Tubes were inverted 10 times to mix and then placed at 56°C to incubate overnight. After incubation, lysate was manually transferred to an S‐block (Qiagen) and DNA extraction was completed using a QiaCube HT robotic workstation and QIAamp 96 QIAcube HT kit according to the manufacturer's recommendations with two minor modifications: TopElute was not used and final elution was performed with 90 μL of buffer EB instead of 200 μL of Buffer AE.

### 2.3 Determining Potato Psyllid Mitochondrial Haplotypes

We used high‐resolution melt (HRM) analysis to determine the mitochondrial haplotype of each individual psyllid, as described in (Swisher et al. 2012, 2014). For any individuals producing ambiguous HRM results and all individuals in populations where multiple mitochondrial haplotypes were detected, results were confirmed with Sanger sequencing.

### 2.4 Screening for ‘Ca. Liberibacter Solanacearum’ and Multi‐Locus Sequence Typing

To screen each individual psyllid for CLso, we performed PCR with the primers rp01‐F/‐R and Phusion HF DNA polymerase (ThermoScientific) as described in (Kenney et al. 2024). After gel electrophoresis, we excised amplicons of the expected size to purify and send for Sanger sequencing (Retrogen Inc.). We used BLASTn to search each resulting sequence against the NCBI database to confirm that it originated from CLso and make preliminary variant assignments. For one sample of each CLso variant from each potato psyllid population that tested positive for CLso, we then amplified, sequenced, concatenated and aligned seven additional multi‐locus sequence typing (MLST) loci as described in (Haapalainen et al. 2018) and (Kenney et al. 2024). Finally, we used the IQTree web server to generate a maximum likelihood tree (1000 bootstraps) based on this alignment.

### 2.5 ddRADseq Library Preparation, Sequencing and Analysis

Libraries were prepared following (Scarparo et al. 2023), which was modified from (Brelsford et al. 2016). Briefly, we digested 7.9 μL of DNA in solution (instead of 6 μL, as in (Scarparo et al. 2023)) from individual psyllids using the restriction enzymes MseI and PstI. We then ligated a universal MseI adapter and a uniquely barcoded (well‐specific) PstI adapter to each sample. After ligation, we used Mag‐Bind Total Pure NGS beads (Omega Bio‐TEk) at a ratio of 0.8:1 (beads: sample solution) to remove smaller fragments from each sample. Next, we performed PCR on each sample in quadruplicate (to minimize PCR bias) using indexed Illumina primers to add a second, plate‐specific barcode to each sample. For each reaction we used 1.325 μL ddH_2_O, 1 μL Q5 buffer, 0.04 μL 25 mM dNTPs, 0.335 μL primer mix, 1 μL Q5 High GC Enhancer, 0.05 μL Q5 Hot Start DNA Polymerase, and 1.25 μL of purified restriction‐ligation DNA (5 μL total reaction volume), and the following program (cover temp: 105°C): 98°C for 30 s followed by 20 cycles of 98°C for 20 s, 60°C for 30 s, 72°C for 40 s and a final elongation step of 72°C for 2 min. After pooling the four sets of PCR products for each sample and subjecting them to one more cycle of PCR, we used gel electrophoresis to check for successful amplification of each sample. We pooled all PCR products from successfully amplified samples by plate and performed a second Mag‐Bind bead purification using the same 0.8:1 ratio as above. Finally, we used a Qubit 3.0 fluorometer (Thermo Fisher) to measure the concentration of each pooled plate of samples, standardized the concentrations, and combined all of the plates of samples into one library. This library was sent to the UC San Diego IGM Genomics Center and sequenced using a 150 base pair paired‐end configuration on an Illumina NovaSeq 6000 (Illumina, San Diego). Sequence analysis followed methods described in Scarparo et al. (2023) and is summarized in the Methods in Supporting Information.

### 2.6 Genetic Diversity

In VCFtools, we used the *–weir‐fst‐pop* option to calculate mean pairwise Weir and Cockerham weighted *F*_ST_ values between different populations, as well as between individuals from the same population sampled at different times (Danecek et al. 2011). To assess genetic differentiation between psyllids sampled on different host plants from the same geographic area and between psyllids sampled on the same host plant from different localities, we calculated and compared *F*_ST_ values for a subset of the data that included psyllids sampled from 18 paired sites (9 pairs), with each pair consisting of a site with *S. umbelliferum* and a geographically adjacent site with a solanaceous crop (Figure S1, Table S1). All sites were sampled once in 2022 and 8/9 site pairs yielded ≥ 7 individuals. One site pair from San Diego county only yielded four and six individuals, respectively, but this pair was included in analyses (unless otherwise specified) because 4–6 individuals are still sufficient given the large number of SNPs generated by the ddRADseq method (Table S1) (Qu et al. 2020). To assess genetic differentiation over time within sites, we focused on another subset of the data that consisted of potato psyllids from all sites with ≥ 7 individuals that we were able to sample twice, once early and once late in the 2022 growing season (Table S1). All five of these populations were associated with solanaceous crops. At two of these locations (UCR Agricultural Operations [UCR Ag Ops] and UCI South Coast REC [UCI SCREC]), psyllids were sampled from potatoes early in the growing season, but from peppers at the same location late in the growing season. To assess genetic differentiation between psyllid populations on a crop (potato) and desert host plants (*Lycium* spp.) we focused on a subset of the data from all sites in the California desert region yielding ≥ 7 psyllids (Table S1). Finally, we used VCFtools to perform windowed *F*_ST_ analyses between pairs of entire mtCOI haplotype groups (Northwestern vs. Southwestern, Southwestern vs. Western and Northwestern vs. Western; we excluded the Central haplotype, because we had very few individuals) using the *–weir‐fst‐pop* option with *–fst‐window‐size* 50,000 and *–fst‐window‐step* 50,000 (Danecek et al. 2011). We visualized the results using the *manhattan* function in the *qqman* package (Turner 2018) in R v4.3.0 (R Core Team 2023).

### 2.7 Population Structure

We used PLINK v1.90b6.25 (Purcell et al. 2007) to perform a principal component analysis (PCA) on the entire dataset of 111,587 SNPs across 590 individual psyllids. We then used the function *ggplot* (Wickham 2009) in R to plot principal components one and two. Clustering was the same (not shown here) with both the entire dataset of 111,587 SNPs and a dataset that underwent linkage disequilibrium pruning using PLINK, so we used the entire dataset for all further analyses. Because individuals clustered very strongly by mtCOI haplotype in our initial PCA, we also performed separate PCAs using just the individuals of each mitochondrial haplotype group to investigate population structure within each of these groups. Additionally, we calculated the proportion of shared alleles between all pairs of individuals using *–distancesquare 1‐ibs* option in PLINK and constructed an unrooted neighbor‐joining tree using these pairwise genetic distances in MEGA (Kumar et al. 2024). For visualization purposes, we included only a subset of 209 individuals in our tree; this subset was made up of eleven pairs of populations from crops and native Solanaceae from throughout the state of California. To further visualize the relationships among haplotypes, mtCOI sequences were converted to the NEXUS format (Maddison et al. 1997), and a haplotype network was constructed using the TCS algorithm implemented in PopArt version 1.7 (Leigh and Bryant 2015). We also used ADMIXTURE v. 1.3.0 (Alexander et al. 2009) to characterize and visualize the genetic structure of potato psyllid populations and admixture of individuals. For a limited number of samples from this study that underwent whole genome sequencing for a separate project (on CLso effector genes), we were able to assemble the genomes of *Candidatus* Carsonella ruddii, the main obligate nutritional endosymbiont (Table S6). As an additional line of evidence, we analyzed *Carsonella* diversity among the three identified psyllid lineages using SNP‐based clustering analysis and calculations of the Average Nucleotide Identify (ANI) between *Carsonella* genomes of each individual (Methods in Supporting Information) (Jain et al. 2018).

## 3 Results

### 3.1 Distribution of Psyllid mtCOI Haplotypes by Habitat

In total, we collected and sequenced 639 individual potato psyllids from 50 populations spanning the state of California (Figure 1A) along a transect from Yolo County in the north to San Diego County in the south—approximately 835 km (Figure 1B, Table S1). Of the 283 potato psyllids sampled from *Solanum umbelliferum* , mtCOI sequencing revealed that all but four individuals were of the Northwestern haplotype; two, from the Barrel Spring population in San Diego County, were of the Southwestern haplotype, and two, from the Dawson population in San Diego County, were of the Western haplotype (Figure 1C). In both of these populations, the Northwestern haplotype was also present and made up the majority of individuals. We also collected two potato psyllids from another native nightshade, *Solanum douglasii* , in Los Angeles County, and these were also of the Northwestern haplotype (Table S1).

**FIGURE 1:**
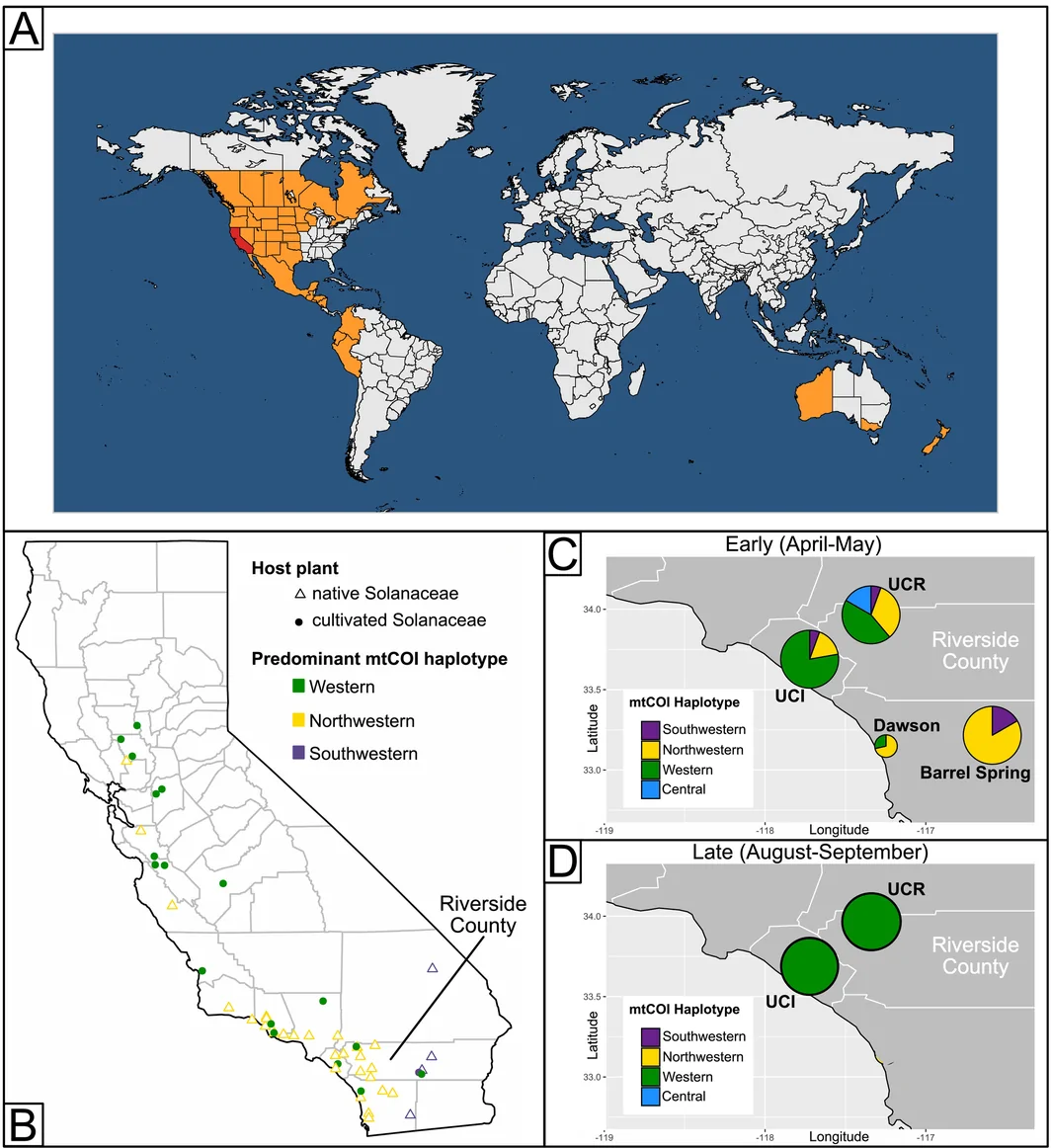
(A) Global distribution of *Bactericera cockerelli* as of June 2026 (EPPO database). California is shown in red to highlight the study area relative to the global range. (B) Predominant mtCOI haplotype of 49 potato psyllid populations sampled throughout California on wild vs. cultivated host plants from spring 2022 to spring 2023. The orange line divides the desert ecoregion (right of the line) from other ecoregions that receive more precipitation (left of the line). (C) Close‐up map showing the only 4/49 populations where a mixture of more than one psyllid mtCOI haplotype was detected (hosts sampled: UCR & UCI = potato, Dawson & Barrell Spring = *S. umbelliferum* ). Mixed haplotype populations were only detected during April–May 2022 in the three most southwestern sampled counties of California: Orange, San Diego and Riverside. (D) mtCOI haplotypes present when two of these populations were resampled in August–September 2022 (hosts sampled: UCR & UCI = pepper).

In 2022, we sampled a total of 336 potato psyllids from cultivated Solanaceae, 264 of which were collected from pepper, including five individuals from one commercial field in the Coachella Valley desert (Table S1). All potato psyllids collected from pepper were of the Western haplotype. We collected just seven potato psyllids from tomatoes; these were also of the Western haplotype. We collected a total of 65 potato psyllids from potatoes. Three of these populations, Zuckerman in San Joaquin County, Pinnacle in San Benito County and Allied in the high desert of Los Angeles County, yielded only Western haplotype psyllids. The remaining two, sampled early in the season (May) at UCR Ag Ops in Riverside and UCI SCREC in Orange County, were majority Western haplotype, but with other haplotypes present in lower numbers. At UCI SCREC, three Northwestern haplotype psyllids and one Southwestern haplotype psyllid were collected, and at UCR Ag Ops, six Northwestern haplotype, one Southwestern haplotype, and three Central haplotype psyllids were collected (Figure 1C). The Central haplotype has not previously been detected in California. When these two populations were sampled again at the end of the growing season (when potatoes had been replaced by pepper), we only collected Western haplotype potato psyllids (Figure 1D).

In 2023, we collected 26 potato psyllids from the native desert Solanaceae *Lycium andersonii* and eight individuals from *Physalis crassifolia* (Table S1). All of these psyllids were of the Southwestern haplotype. We also sampled 18 psyllids from one commercial potato field in the California desert in 2023; all of these psyllids were also of the Southwestern haplotype.

### 3.2 Potato Psyllid Population Structure

We retained a total of 111,587 SNPs in 590 individual potato psyllids for population genomics analyses: 286 Northwestern haplotype, 247 Western haplotype, 54 Southwestern haplotype and 3 Central haplotype. Principal component analysis of all 590 individuals revealed clustering by mtCOI haplotype (Figure 2). Northwestern haplotype potato psyllids (almost all from *Solanum umbelliferum* ) formed one cluster, Western haplotype potato psyllids (virtually all from cultivated Solanaceae) formed a second cluster, and Southwestern haplotype potato psyllids (from both cultivated and native Solanaceae, nearly all sampled in the desert in 2023) formed a third distinct cluster. Less than 5% (~25/590) of individuals did not fall neatly into one of these three clusters, including the only three Central haplotype individuals we detected; the majority of these outlier individuals were from the few populations and sampling time points where we detected mixtures of individuals of different mtCOI haplotypes (UCR Ag OPS and UCI SCREC, as well as Barrel Spring), and, thus, may be the result of interbreeding between individuals with different mtCOI haplotypes. PCA analysis conducted using > 4000 high‐quality biallelic SNPs from *Carsonella* genomes of Northwestern, Western and Southwestern haplotypes revealed similar clustering patterns to those detected in the analysis of host nuclear genomes (Figure S2).

**FIGURE 2:**
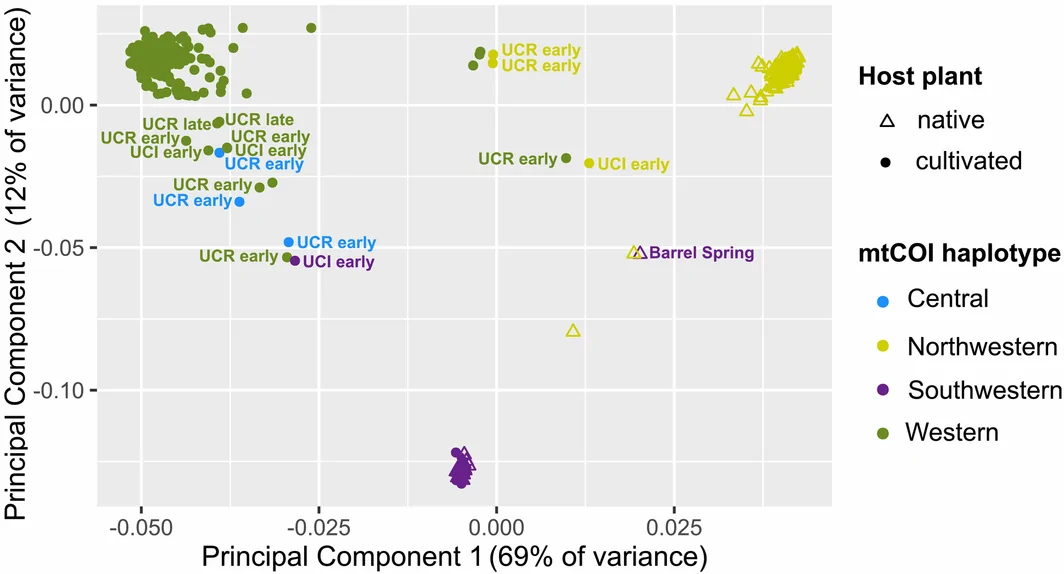
PCA plot of genetic variation between all 590 individual psyllids from 49 populations, based on 111,887 SNPs.

In addition to PCA, we built a neighbor‐joining tree (Figure 3), performed a mtCOI haplotype network analysis (Figure 4A), and conducted an ADMIXTURE analysis to visualize inferred genetic relationships between potato psyllid populations in California (Figure 4B). In these analyses, potato psyllids clustered by mtCOI haplotype, with just a handful of exceptions, primarily from the four early season mixed‐haplotype populations identified via mtCOI haplotyping (UCI, UCR, Dawson and Barrel Spring). We also detected clustering by host plant type (wild vs. cultivated) that aligned with clustering by mtCOI haplotype (Figure 3). Nearly all psyllids in the Northwestern haplotype cluster were collected from wild solanaceae, while the opposite was true for individuals in the Western haplotype cluster. The Southwestern haplotype contained a mixture of individuals collected from wild and cultivated hosts.

**FIGURE 3:**
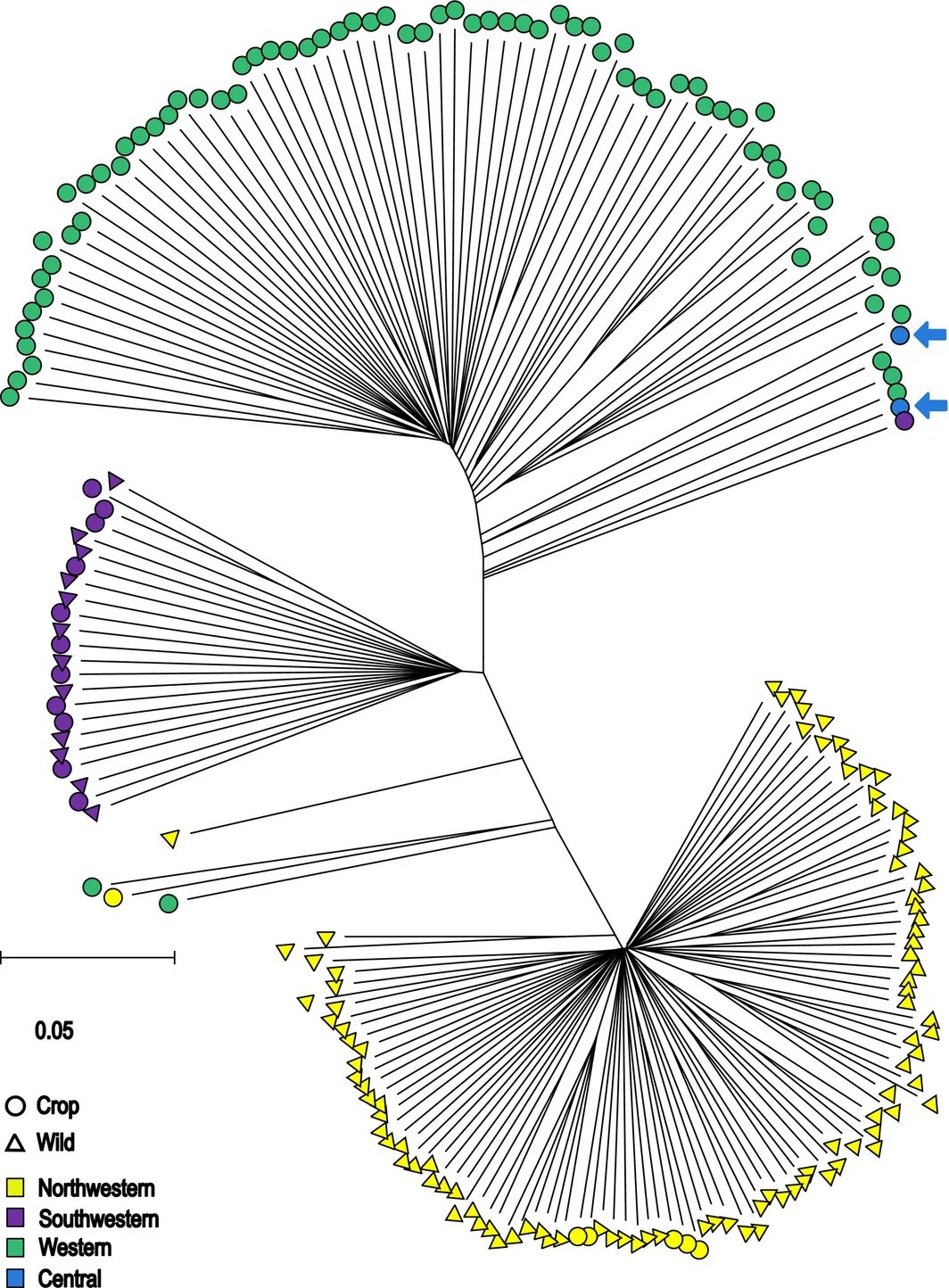
Neighbor‐joining tree depicting inferred relationships between 209 individual potato psyllids with < 10% missingness, sampled from 11 pairs of populations from crop and native host plants throughout California. Blue arrows point out the two Central haplotype individuals included in the analysis. The tree is based on 111,857 SNPs characterized via ddRADseq. Note that branch lengths within groups are artificially elongated due to use of SNP data to calculate distances. SNP filtering excludes sites without variation, leading to retention of only variable positions and augmented estimates of genetic distance between individuals.

**FIGURE 4:**
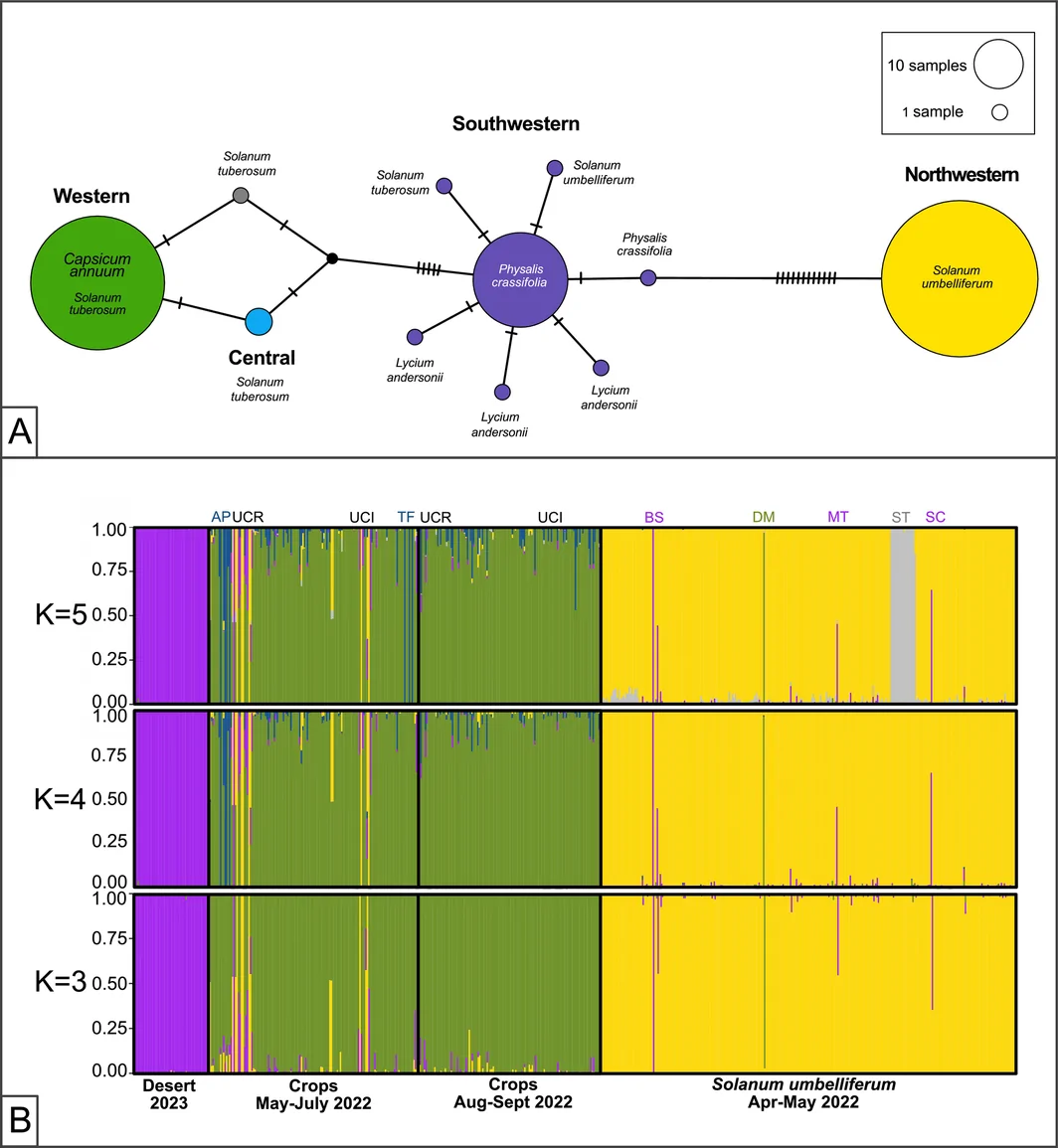
(A) Haplotype network showing potato psyllid genetic structure in California based on the mitochondrial gene COI. Each circle represents a unique mtCOI sequence variant, and circle sizes are proportional to the number of individuals of each variant. Colors represent three putative cryptic species, with Central haplotype individuals tentatively grouped as the same species as the Western haplotype. Haplotypes differing from each other by one base pair are separated by a single line, and ticks along the line represent additional base pair differences. (B) Stacked bar plot showing population structure of California potato psyllids based on ADMIXTURE analysis of all 590 individuals from 49 sampling sites. *K* = 3 CV error: 0.46659, *K* = 4 CV error: 0.46715, *K* = 5 CV error: 0.46564. Inferred ancestry closely matched mitochondrial haplotype (purple = Southwestern, green = Western, yellow = Northwestern). However, within the Northwestern haplotype group the Stebbins population (ST) was unique from all other populations (grey), and within the Western haplotype group the Allied Potato (AP) and early season Talley Farms (TF) populations contained several individuals that were more similar to each other (blue) than any other populations.

The mtCOI network analysis was congruent with the results of our neighbor‐joining tree and showed that most psyllids clustered into the previously described Northwestern, Southwestern and Western haplotypes (Figure 4A). These three lineages are differentiated from each other by 10–13 base pairs. An additional nine individuals clustered separately from these three primary haplotypes, differing from either the Southwestern or Western haplotype by just two base pairs. This included three individual psyllids with mitochondrial COI sequences identical to the previously described Central haplotype.

The ADMIXTURE analysis, performed on all 590 individuals from 49 populations, identified *K* = 5, with a cross validation (CV) error of 0.46564, as the most probable number of genetic groups, but only by a small margin over *K* = 3 and *K* = 4 (CV errors of 0.46659 and 0.46715, respectively). The primary three genetic groups correspond with the Northwestern, Southwestern and Western mitochondrial haplotypes (Figure 4B). Within the Northwestern group, the Stebbins population was distinct from the rest, and within the Western haplotype group, some individuals of the early season Talley Farms (TF) and Allied (AP) populations were more similar to each other than any other individuals (Figure 4B). The populations that showed evidence of having a few individuals with mixed ancestry in the principal component analysis (UCR AgOps, UCI SCREC and Barrel Spring, Figure 2) also showed evidence of mixed ancestry in the ADMIXTURE analysis (Figure 4B).

We also performed PCA analyses to look for nuclear genetic structure within each of the mtCOI haplotype groups (Figure S3). Within the Northwestern mtCOI haplotype, several populations, in particular the two northernmost, Stebbins and Alum Rock, formed distinct clusters from the rest (Figure S3A). In the case of the Western haplotype, many of the individuals from the two early season mixed‐haplotype populations, UCR Ag Ops and UCI SCREC potato, separated out from the rest, but without forming a clear cluster (Figure S3B). Some of the individuals from the high desert potato population, Allied, also did not cluster with the rest (Figure S3B). For the Southwestern haplotype, two outliers from Grant potato and JTNP *Physalis crassifolia* did not cluster neatly with the rest, but otherwise there was no distinct pattern of genetic differentiation between any of the populations sampled (Figure S3C).

### 3.3 Potato Psyllid Genetic Diversity

We detected high *F*_ST_ between neighboring pairs of potato psyllid populations from different hosts (crop vs. native; mean of 0.339), and lower *F*_ST_ between more geographically distant pairs of populations from the same type of host (mean of 0.0334 for crops and 0.0679 for *S. umbelliferum* ) (Figure 5). However, in the southern California counties of Orange, Riverside and San Diego, where we detected some mixing of mtCOI haplotypes on both crops and native nightshades early in the season, pairwise *F*_ST_ between crop‐ and native‐nightshade‐associated populations was lower (mean of 0.23) than the mean value for populations from the northern collection areas (mean of 0.394).

**FIGURE 5:**
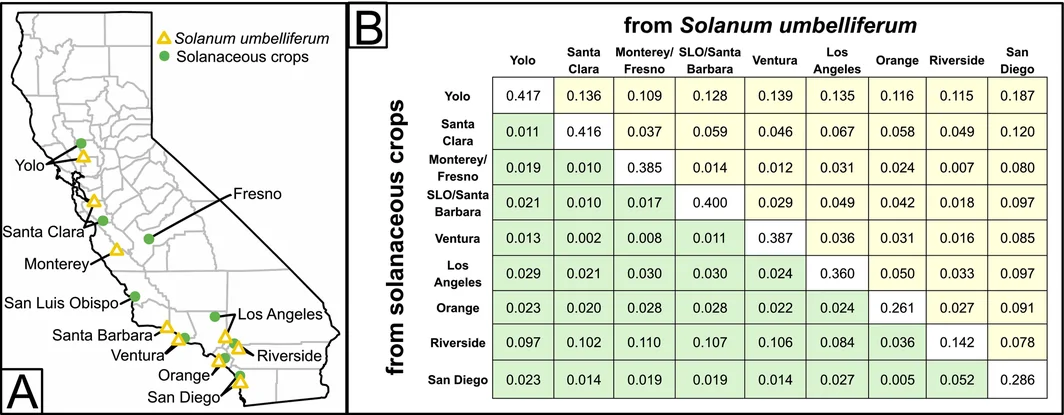
(A) Geographically paired sampling sites with wild and cultivated hosts. (B) Pairwise Weir and Cockerham weighted *F*_ST_ values for one psyllid population from *Solanum umbelliferum* (above the diagonal) and the closest population from solanaceous crops (below the diagonal) in each county or pair of counties. Psyllid samples from crops in Santa Clara, Ventura, Orange and Riverside Counties were sampled early in the season (May–July) and again late in the season (August–September). Only early season samples were used here, as they were collected closer in time to the samples from *S. umbelliferum* (April–May).

When comparing *F*_ST_ of crop‐associated populations early in the 2022 growing season to *F*_ST_ later in the season (Figure 6A,B), we found elevated *F*_ST_ between the mixed haplotype populations (UCR Ag Ops and UCI SCREC potato) and all other populations early in the season (means of 0.1029 and 0.088, respectively, compared to 0.0018 among all other populations), but very low *F*_ST_ between all populations late in the season (≤ 0.0092, or a mean of 0.00636; Figure 6B).

**FIGURE 6:**
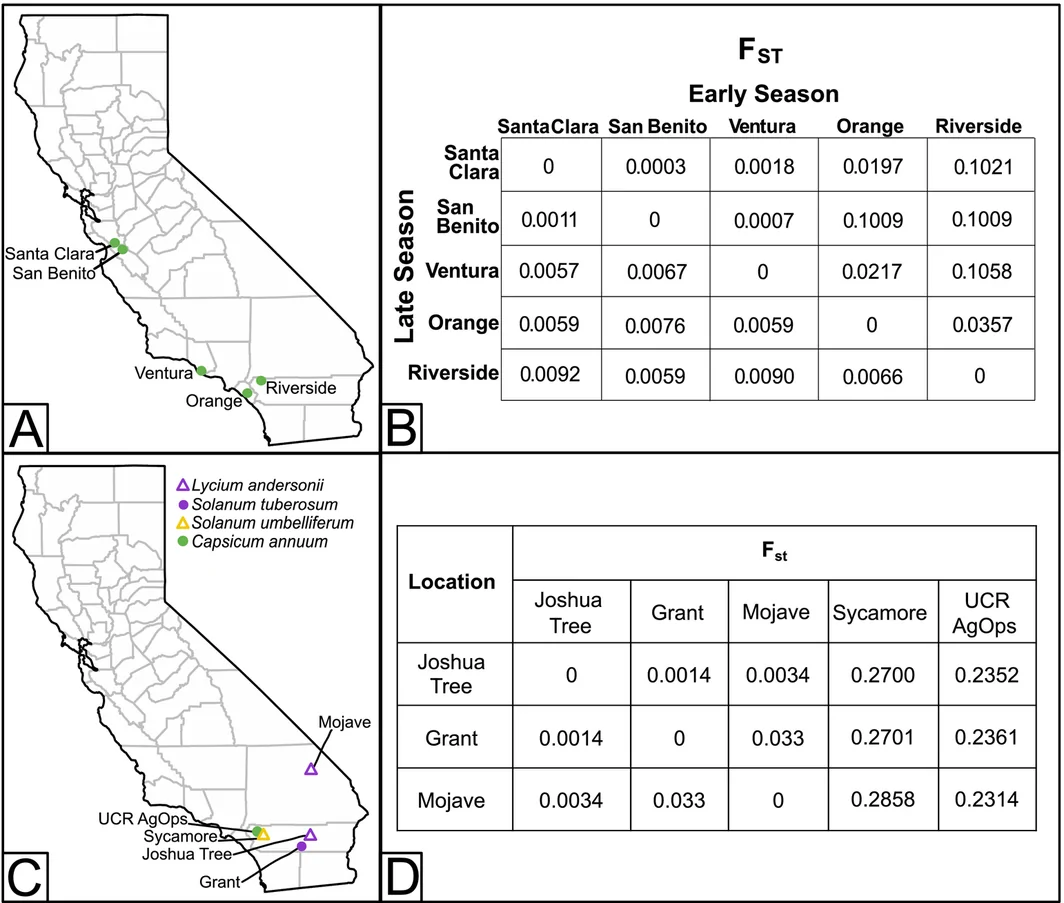
(A) Map showing locations where potato psyllids were sampled from agricultural fields at multiple time points: Once early in the growing season (May–July) and again late in the growing season (August–September), labeled by county. (B) Pairwise Weir and Cockerham weighted *F*_ST_ of crop‐associated potato psyllid populations sampled early in the growing season (above the diagonal) and late in the growing season *F*_ST_ (below the diagonal). (C) Map showing locations of Southwestern haplotype potato psyllid populations from which we sampled ≥ 8 individuals in the California desert in February–March of 2023. (D) Table showing pairwise Weir and Cockerham weighted *F*_ST_ of each population compared with all other desert 2023 populations and each population compared with the geographically nearest site with Northwestern haplotype psyllids (from *Solanum umbelliferum—*Sycamore) and Western haplotype psyllids (from *Capsicum annuum* —UCR AgOps).

For desert potato psyllid populations sampled in 2023 (Figure 6C,D), *F*_ST_ between these populations was low (mean of 0.0126). When compared with the nearest populations from *S. umbelliferum* (Sycamore) and crops (UCR Ag Ops, early season potato), *F*_ST_ was much higher, at ≥ 0.27 and ≥ 0.23, respectively.

We also compared genetic diversity between mtCOI haplotype groups (Figure 7). Sliding window analysis revealed that *F*_ST_ between the Northwestern and both other haplotypes is consistently high across all 13 chromosomes of the potato psyllid genome, except for the ends of the chromosomes (Figure 7A,B). This suggests that populations have fixed differences at most SNPs (maximum weighted *F*_ST_ was as high as 0.9670 between Northwestern and Western, and 0.9550 between Northwestern and Southwestern, for some windows). Levels of genetic differentiation between the Western and Southwestern haplotype groups were not quite as consistent across the entire genome (Figure 7C), but the maximum weighted *F*_ST_ was still very high, at 0.88. These levels of genetic differentiation are supported by the outputs of pairwise ANI calculations for *Carsonella* genomes of individuals of the Northwestern, Western and Southwestern haplotypes (Figure S4). The lowest ANI values (indicating the most differentiation) were calculated for Northwestern‐Western comparisons (97.78–97.94). The next highest were for Northwestern‐Southwestern comparisons (97.99–98.16), and Western‐Southwestern comparisons yielded the highest inter‐haplotype values (98.72–98.85). Within‐haplotype comparisons all exceeded 99.45.

**FIGURE 7:**
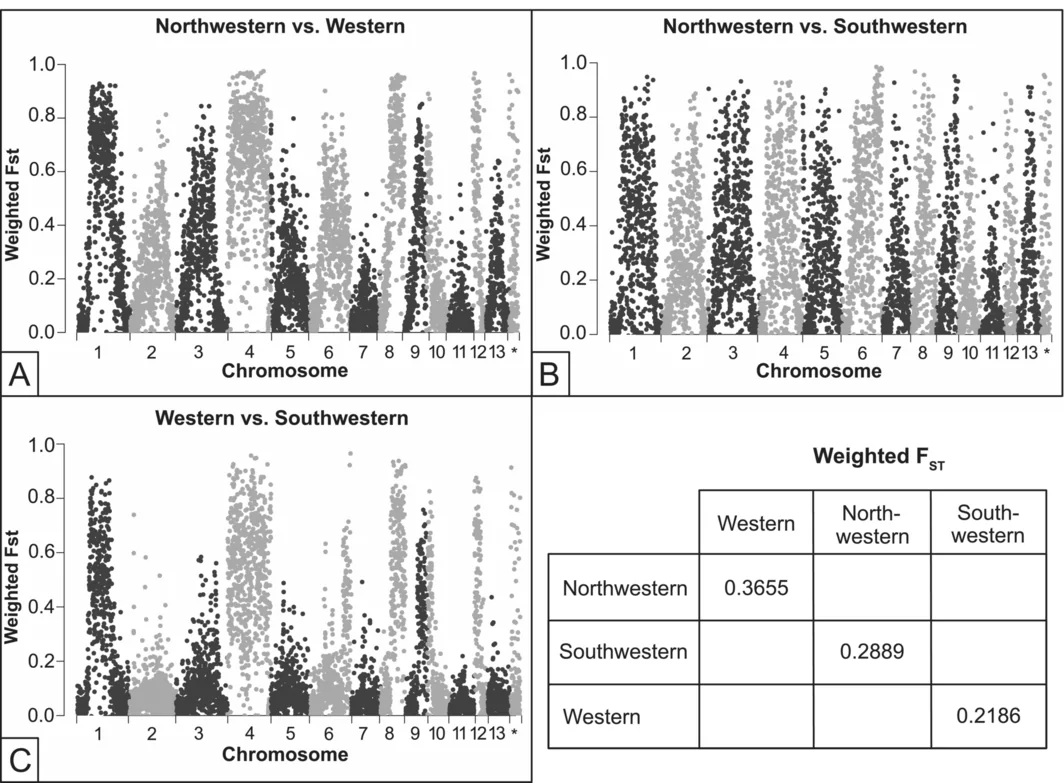
Variation in levels of differentiation as evaluated using sliding window Weir and Cockerham *F*_ST_ analysis across the thirteen chromosomes of the potato psyllid genome between (A) all Northwestern and all Western haplotype individuals, (B) all Northwestern and all Southwestern haplotype individuals, and (C) all Western and all Southwestern individuals. The asterisk in place of chromosome 14 denotes a compilation of all unassembled scaffolds. The inset table shows the overall Weighted Weir and Cockerham pairwise *F*_ST_ for each comparison of haplotypes.

### 3.4 CLso Diagnostics and MLST

We detected CLso in 13 out of 49 of the potato psyllid populations that we sampled (Figure 8) and an overall total of 39 out of 639 individual potato psyllids tested (6.1%). All populations with infected individuals were located in southwestern California, in the Mediterranean to semi‐arid region closer to the coast. Only two populations with infected individuals were found in association with crops: Deardorff, on pepper in Ventura County, and West Coast, on Tomato in San Diego County (Table S1). The other 11 populations were associated with *Solanum umbelliferum* . 50S and MLST sequencing and phylogenetic analysis revealed a total of five CLso variants infecting these potato psyllid populations: A, B, F, G and Sumb2 (Figure 8, Figure S5). CLso B, typically considered the most virulent, crop‐associated variant, was the most widespread; it was detected in six populations associated with *S. umbelliferum* across five counties (Santa Barbara, Ventura, Los Angeles, Riverside and San Diego) and the San Diego County population associated with tomato (West Coast). In two of the *S. umbelliferum* ‐associated populations in which we detected CLso B, Sycamore in Riverside and Barrel Spring in San Diego County, CLso Sumb2 was also present, sometimes coinfecting the same individual psyllid. CLso Sumb2 was previously only detected in *S. umbelliferum* and associated potato psyllid populations in southwestern Riverside County (Kenney et al. 2024). In this study, we detected Sumb2 in a third population of psyllids associated with *S. umbelliferum* in Riverside County.

**FIGURE 8:**
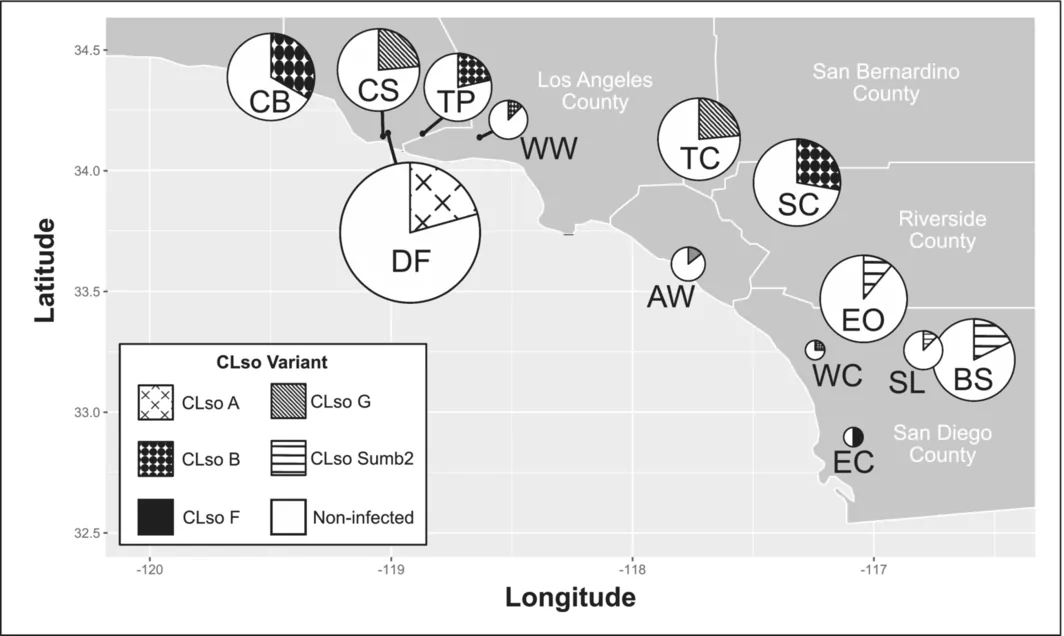
Map of southwestern California showing CLso prevalence and variants in the 13/49 potato psyllid populations that tested positive for CLso infection. The size of the circle is proportional to the total number of psyllids tested from each population (see Table S2). Location codes and mtCOI haplotypes of psyllids from each site: AW, Aliso Woods (NW); BS, Barrel Spring (NW); CB, Carpinteria Bluffs (NW); CS, CSU Channel Islands (NW); DF, Deardorff Farms (W); EC, Elliot Chaparral Reserve (NW); EO, Emerson Oaks Reserve (NW); SC, Sycamore Canyon Park (NW); SL, San Luis Rey turnout (NW); TC, Thompson Creek (NW); TP, Triunfo Park (NW); WC, West Coast Tomato (W); WW, Wild Walnut Park (NW).

Another recently discovered variant, CLso G, was detected in three *S. umbelliferum‐associated* potato psyllid populations across three counties, Ventura, Orange and Los Angeles, but not from any of the populations sampled in Riverside County where plants infected with this variant were previously found (Kenney et al. 2024). CLso F, known only from a single Zebra chip‐symptomatic potato tuber grown in Oregon in 2017 (Swisher Grimm and Garczynski 2019), was detected in 2/4 individual potato psyllids collected from *Solanum umbelliferum* at the Elliot Chaparral reserve in San Diego County, but in no other populations. All of the *S. umbelliferum* ‐associated potato psyllids that tested positive for CLso were of the Northwestern haplotype, and all of the CLso‐positive psyllids associated with crop plants were of the Western haplotype (Table S2).

## 4 Discussion

Biological and behavioral differences among cryptic species may underlie complex pathogen dynamics that make it difficult to predict and manage disease in crops (Wosula et al. 2017; Milenovic et al. 2019; Campbell et al. 2023) or execute conservation programs (Bickford et al. 2007). Efforts to reveal the hidden biodiversity of insect vectors are essential to understand the factors driving their distribution and abundance (such as host plant preferences) and how these factors subsequently shape the prevalence of the pathogens they transmit. A previous study documented patterns of temporal separation and spatial separation by host plant and geographic area among morphologically indistinguishable mtCOI haplotypes of potato psyllid vectors in the Southwest (Kenney et al. 2024). These haplotypes also showed distinct associations with novel genetic variants of the plant pathogen, *Candidatus* Liberibacter solanacearum, that had not previously been detected in crops (Mauck, Sun, et al. 2019; Kenney et al. 2024). These lines of evidence, as well as others documenting mating incompatibility between some haplotypes (Cooper et al. 2015; Mustafa et al. 2015), led us to hypothesize that the potato psyllid may be a complex of several cryptic species.

Through a large‐scale sampling effort of solanaceous crops and wild plants in a region of the U.S. where all four known potato psyllid mtCOI haplotypes occur (California, USA), we found evidence that *B. cockerelli* may consist of at least three distinct species, represented by the Western haplotype and, tentatively, the Central haplotype (included based on limited sampling, hereafter, Lineage WC), the Southwestern haplotype (hereafter, Lineage SW), and the Northwestern haplotype (hereafter, Lineage NW) (Figure 4). ddRADseq confirmed very high levels of average *F*_ST_ between these lineages even when they were sampled from adjacent populations (Figure 5). In contrast, members of the same lineage had much lower levels of *F*_ST_, even when sampled from geographically distant populations. We also found high levels of *F*_ST_ across large regions of all 13 chromosomes in every pairwise comparison among the three lineages (Figure 7).

For a subset of samples used in this study, additional sequencing data were available to explore lineage‐specific variation in the vertically‐transmitted obligate symbiont, *Candidatus* Carsonella ruddii (Methods in Supporting Information, Figures S2 and S4, Table S6). Analysis of 4326 SNPs across the *Carsonella* genomes revealed clear separation of Lineage NW, WC and SW and mirrored the population structure found in host RADseq and mtCOI datasets (Figure S2). More striking are the calculated ANI values for inter‐lineage *Carsonella* comparisons, which are as low as 97.78 (Lineage NW vs. Lineage W). Other studies exploring diversity of the primary symbiont of whiteflies (*Candidatus* Portiera aleyrodidarum) report ANI values that are higher than 97% for comparisons between known cryptic species (Campbell et al. 2023; Wang et al. 2025). For example, Campbell et al. (2023) found that multiple sympatric cryptic whitefly species in the sub‐Saharan Africa clade (SSA) have > 7% divergence in mtCOI sequences and exhibit reproductive incompatibility, but ANI values for their *Portiera* symbionts in pairwise comparisons are > 99%. ANI values for *Portiera* comparisons between individuals of allopatric cryptic whitefly species with even greater mtCOI divergence (19%–23%) are also greater than or equal to 97% (Campbell et al. 2023). These studies provide a baseline for interpreting a standard divergence estimate used for species delimitation in free‐living prokaryotes (ANI) (Jain et al. 2018) as applied to obligate symbionts (Campbell et al. 2023). Interpreted in this context alongside clustering analysis (Figure S2), *Carsonella* divergence patterns further support our hypothesis that the three potato psyllid lineages may be cryptic species.

High genome‐wide *F*_ST_, principal component analysis, and ADMIXTURE analysis all suggest the three lineages are experiencing reproductive isolation. We rarely detected putative hybrids, and when we did, these individuals were only recovered from sites with a brief period of spatial and temporal overlap among lineages (UC Riverside and UC Irvine agricultural experiment stations and the Barrel Springs site) (Figure 1). At the UC sites, we detected possible hybridization between Lineage WC and Lineage SW, and between Lineage WC and Lineage NW, but only for a brief period in May; later sampling in these sites yielded only Lineage WC psyllids. At the Barrel Springs site, we detected possible hybridization between Lineage SW and Lineage NW. To confirm introgression patterns, further sampling with greater temporal resolution would have to be performed at these sites, followed by additional genomic analyses (e.g., the *D3* test, Hahn and Hibbins 2019). However, our analyses thus far suggest hybridization may be uncommon and backcrossing rare or absent (Figures 2 and 4). This aligns with a previous study performed with psyllids collected in the Pacific Northwest, which found that matings between Lineage WC females and Lineage NW males are possible, but result in reduced egg viability, while matings between Lineage NW females and Lineage WC males produced no viable eggs (Mustafa et al. 2015). Follow‐up studies on populations of these lineages in the Pacific Northwest suggest that incompatibility may be partially driven by presence or absence of *Wolbachia* symbionts, and reinforced by spatial separation among lineages due to different host plant preferences (Cooper et al. 2015; Fu et al. 2020). Our results are consistent with partial reproductive incompatibility observed in these studies.

Lack of putative hybrids in late season sampling and high *F*_ST_ levels across the genome suggest that rare hybrids produced from matings between members of the different lineages in California may not be able to reproduce or persist in the environment (Figures 1 and 7). Future work could test this hypothesis by evaluating the viability of offspring from Lineage WC female × Lineage SW or Lineage NW male crosses. It is also interesting that putative hybrids were mostly observed at two highly diversified semi‐agricultural sites (university agricultural research stations) featuring small patches of irrigated crop plants, often not managed for pests, among abundant weedy and unmanaged patches. These unique habitats may favor spatial and temporal overlap among lineages that are otherwise strongly separated by host plant preferences and geography.

Spatial and temporal overlap among the three lineages in the presence of diverse weeds and crop plants may also create pathways for CLso exchange between different psyllid species and adaptation to new plant hosts (Borges et al. 2017; Thapa et al. 2020). In the same areas where we found spatial and temporal overlap of psyllid lineages, we detected the most CLso diversity, although overall prevalence of CLso infection across psyllid lineages was quite low (6.1%). Even with this low prevalence, there were five unique CLso variants present in the region, sometimes occurring as coinfections in the same individual psyllids and host plants (e.g., CLso B and Sumb2 in *S. umbelliferum* and associated psyllids; Kenney et al. 2024). This diversity could lead to the psyllid lineages acquiring new CLso symbionts, as transmission of CLso between different psyllid lineages is possible through local movement of the bacteria between adjacent individuals feeding on the same plant (Borges et al. 2017). Multiple infections in plants or psyllids may even facilitate genetic exchange among CLso variants or CLso and other symbionts (Nakabachi et al. 2013; Tan et al. 2021). Such exchanges may have contributed to the evolution of endophytic and pathogenic traits common to the *Ca*. Liberibacter lineage (Tan et al. 2021). Our results already provide evidence that new pathogenic variants are present in this area. We collected two Lineage NW psyllids infected with CLso F, a pathogenic variant that was first described causing disease in potatoes in Oregon in 2019 (Swisher Grimm and Garczynski 2019) (Table S2). Our MLST analysis showed that this variant is very closely related to CLso G (Figure S5), which infects native *S. umbelliferum* and Lineage NW (Kenney et al. 2024). CLso F may be a variant of CLso G that has evolved the ability to infect potatoes. The fact that it has not spread to cause widespread disease in potatoes could be due to maintenance of an association with Lineage NW which does not occur on crop hosts. These hypotheses can be further explored through comparative analyses of the genomes of CLso G and F and through continued sampling of crop and non‐crop habitats to monitor CLso diversity and link CLso variant identity with disease symptoms and psyllid lineages.

While a small region along the coast supports some temporal and spatial overlap among the three psyllid lineages identified in our study, our broader collection efforts provide clear evidence that these lineages differentiate by region and host plant preferences. Throughout most of the state of California, psyllid movement between native and cultivated Solanaceae is relatively rare. In Central and Northern California, we detected only Lineage WC psyllids on crops and only Lineage NW on native *Solanum umbelliferum* , with little evidence of psyllid movement or gene flow between natural and crop environments. Even in coastal southern California this pattern was largely maintained, with the exception of the UCI and UCR research stations discussed above, where we detected movement of Lineage WC and Lineage NW between natural and crop environments during the late spring. Finally, in the inland desert region of California, neither Lineage WC nor Lineage NW psyllids were present in large numbers. However, Lineage SW was collected from both native and cultivated Solanaceae in this region, apparently moving between these crops and native plants with great frequency, as evidenced by the low levels of genetic differentiation between crop and native plant‐associated populations (Figures 2 and 4). These results suggest the Lineage SW psyllids have a wider breadth of possible host plants and may be able to use solanaceous crops as oversummering hosts when native *Lycium* and *Physalis* plants are dormant. However, we did not detect any CLso in the Lineage SW psyllids sampled within this region (Table S2), and in previous work, we only detected CLso G (which does not infect crops) (Kenney et al. 2024), so there may be little risk of this lineage transmitting CLso to crops or wild plants.

Unlike Lineage SW, the Lineage NW is strictly limited to its native host, *S. umbelliferum* . We detected only nine Lineage NW psyllids on solanaceous crops throughout California, and only early in the growing season, suggesting that psyllids of this lineage are not oversummering on solanaceous crops when *S. umbelliferum* is dormant. If this lineage is native to California and has been associated with *S. umbelliferum* since before the widespread cultivation of solanaceous crops in this region, it is logical that it would have evolved a strategy for surviving the summer dormancy period of *S. umbelliferum* that does not involve irrigated crops. Other psyllid species diapause as eggs or nymphs on the bare stems or roots of their reproductive host plants (Lal 1934; Loginova 1970, 1976; Mustafa and Najjar 1985; Rapisarda and Others 1990; Lauterer 1993; Wheeler and Rawlins 1993; Hodkinson et al. 1999; Hodkinson 2009). This behavior has never been observed in potato psyllids, but cannot be ruled out because most work on potato psyllid life history strategies involved only cultivated host plants being grown as annuals, rather than wild perennials. There are scattered reports of potato psyllids on non‐solanaceous evergreen plants, including conifers and rabbitbrush, at higher elevations in California in summer and fall (Table S3) (Crawford 1914). These ‘shelter plants’ (Burckhardt et al. 2014) may be the summer host for Lineage NW, and possibly Lineage SW, as they are for other psyllids in the family Triozidae (Valterová et al. 1997; Thébaud et al. 2009; Jarausch and Jarausch 2016). In 2024, after this study was already completed, we opportunistically discovered thousands of potato psyllids on pine trees during the summer (July 26, 2024) at Grass Lake (Lat/Lon: 36.56163, −118.24041; Elevation: 3262 m) on Mount Whitney in the Sierra Nevada mountain range of California. We collected individuals from this site and performed mtCOI haplotyping as described in the methods. All individuals for which sequencing was successful were Lineage SW (Table S4), providing evidence that at least one lineage predominantly associated with winter‐growing wild solanaceae is using conifers for oversummering. Sequencing a portion of the *matK* barcoding gene from the conifer hosting the psyllids suggests the shelter plant species is most likely *Pinus contorta* (lodgepole pine) which is common at high elevations in the Sierra Nevada mountains (Tables S4 and S5).

Lineage WC potato psyllids also have strict host plant preferences, but for crops instead of native Solanaceae. We only recovered two Lineage WC psyllids from a single *S. umbelliferum* plant (at Dawson Los Monos Canyon Reserve, San Diego) in our sampling efforts, and no Lineage WC psyllids were recovered from *Lycium andersonii* or *Physalis crassifolia* . Our finding that Lineage WC psyllids do not use these native host plants supports a previously proposed hypothesis that, despite its name, this lineage was introduced to Western North America from another location, likely the Central U.S. or eastern Mexico (Liu et al. 2006; Fu et al. 2020). The low levels of genetic differentiation that we detected among populations of Lineage WC psyllids (Figure 2), even between Northern and Southern California, also supports the idea that this lineage is a more recent introduction to the region. If Lineage WC is not native to California, it may use some other, still unidentified crop or non‐crop plant species to survive periods when solanaceous crop plant hosts are scarce. In the Pacific Northwest, Lineage WC is also primarily found in crops (potato) but also uses an introduced, ornamental *Lycium* species ( *Lycium barbarum* ) between May and September (Fu et al. 2020) and can overwinter on leaves and stems of the non‐native nightshade, *Solanum dulcamara* (Murphy et al. 2013). However, sequencing the mtCOI gene of wild psyllids overwintering on this host revealed that most are Lineage NW, with only a small proportion being Lineage WC (Swisher, Sengoda, et al. 2013).

While the main overwintering host of Lineage WC in both the Pacific Northwest and the Southwest remains uncertain, the finding that *S. dulcamara* is an overwintering host for Lineage NW in the Pacific Northwest raises an intriguing possibility: invasion of the Pacific Northwest by the non‐native *S. dulcamara* may have created an opportunity for Lineage NW to expand northward from its native range in California, where *S. dulcamara* is largely absent, but *S. umbelliferum* is abundant from Fall through early Spring (Calflora Taxon Reports 7653 and 7664). Fu et al. (2020) also found that Lineage NW uses the non‐native *Lycium barbarum* between May and September in the Pacific Northwest but is absent in potato crops. Thus, two non‐native, non‐crop hosts established in this region are available to support year‐round populations of Lineage NW. A similar range expansion to the Pacific Northwest has likely already occurred for the related species, *Bactericera maculipennis*, which is also native to the Southwest, where it feeds on Convolvulaceae in the genus *Calystegia* (Horton et al. 2019) as well as *S. umbelliferum* (Tran et al. 2024). There is strong evidence that this species was only able to establish in the Pacific Northwest following the introduction and spread of an invasive bindweed species that is a high‐quality host ( *Convolvulus arvensis* ) (Horton et al. 2019). Lineage NW may occur in this region through a similar range expansion facilitated by non‐native invasive plants that provide a new niche for this organism to occupy.

Overall, our extensive sampling efforts revealed that all four mitochondrial haplotypes of the potato psyllid are present in California, including small numbers of the Central haplotype (a haplotype of Lineage WC), which had not previously been detected in this state. Using ddRADseq, we found extremely high *F*_ST_ levels across most of the genome in pairwise comparisons among Western, Northwestern and Southwestern haplotypes, suggesting that these haplotypes may be a cryptic species complex consisting of Lineage WC, Lineage NW and Lineage SW. These results are congruent with divergence patterns of *Carsonella* seen in PCA clustering and pairwise ANI calculations. We also found that putative hybrids are rare and may not persist in the landscape, and that these lineages are strongly separated by host plant preferences for native vs. crop plants. Small regions of habitat near the coast support spatial and temporal overlap among lineages and also house the highest diversity of CLso variants. These include rare variants that we only found associated with Lineage NW in our study (CLso G, CLso Sumb2 and CLso F). One of these variants (CLso F) is pathogenic in potatoes and could be problematic in crops if associations with Lineage WC are possible. Lineage SW and Lineage NW may be native to Southwest, where they are adapted to local native solanaceae and, in some cases, coniferous shelter plants. Presence of Lineage NW in the Pacific Northwest could represent an expansion from its native range in the Southwest, facilitated by introduced, non‐native solanaceae. In contrast, lack of native host plants and overall lower *F*_ST_ values between populations support the hypothesis that presence of Lineage WC in both the Southwest and Pacific Northwest is likely due to an introduction from a more distant location in the Central U.S. or Eastern Mexico. To begin testing these hypotheses, outputs of our local study could be combined with genome‐wide SNP datasets of other local studies (Fu et al. 2020, focusing on the Pacific Northwest) and new datasets generated from sampling efforts in other U.S. states (e.g., Texas, Colorado) and countries (Mexico and Central America). Whole genome sequencing of individuals from these areas can also be used to estimate divergence times and explore variation in genes underlying host plant interactions.

## Funding

Funding was provided by the National Agricultural Statistics Service (CA‐R‐ENT‐5144‐H), the National Institute of Food and Agriculture (2022‐67011‐36635, 2022‐67014‐36271, 2024‐67013‐42769), and the Shipley‐Skinner Reserve‐Riverside County Endowment. The Illumina NovaSeq 6000 machine was purchased by the UC San Diego IGM Genomics Center with funding from a National Institutes of Health SIG grant (#S10 OD026929).

## Conflicts of Interest

The authors declare no conflicts of interest.

## Supporting information


**Figure S1:** Map of California showing 49 locations across 15 counties where potato psyllids were sampled from cultivated (pepper, potato and tomato) and native (*Solanum umbelliferum, S* *. douglasii* , *Lycium andersonii* and *Physalis crassifolia* ) Solanaceae from spring 2022 to spring 2023.
**Figure S2:** Principal component analysis visualizing *Carsonella* SNP variation. PCA was performed using 4326 high‐quality SNPs identified across *Carsonella* genomes from 10 psyllid individuals (Table S6). Circles indicate individuals sampled from crop hosts and triangles indicate individuals sampled from wild hosts. The PCA for *Carsonella* SNPs mirrors the separation and clustering of psyllid haplotypes in the PCA generated from psyllid RADseq SNP data (Figure 2) and the haplotype map (Figure 4), providing an additional line of evidence showing clear lineage differentiation.
**Figure S3:** PCA plots of genetic variation between all potato psyllids of the (A) Northwestern haplotype (289 total individuals), (B) Western haplotype (247 total individuals) and (C) Southwestern haplotype (54 total individuals). Named sites indicate discrete clusters that separate from the main cluster containing individuals from multiple populations. For the Western haplotype, we did not detect distinct clusters of individuals from the UCR Ag Ops and UCI SCREC sites where putative inter‐haplotype hybrids were detected. However, unlabeled points diverging from the main cluster contain many individuals from the early season collections in this site when putative hybrids were present.
**Figure S4:** Heatmap of Average Nucleotide Identities (ANI) between *Carsonella* genomes from individuals of different psyllid haplotypes. Higher ANI values (blues) indicate greater genomic similarity among *Carsonella* symbionts and lower ANI values (reds) indicate dissimilarity. NW = Northwestern, W = Western and SW = Southwestern. Sample codes correspond to information in Table S6.
**Figure S5:** Maximum likelihood tree based on 3917 nt alignment of seven concatenated MLST genes amplified from ‘*Ca*. Liberibacter solanacearum.’ 1000 bootstraps (only values ≥ 70% shown here). Haplotypes are listed on the right. New sequences from this study are in bold text.
**Table S1:** Potato psyllid sampling locations, dates, host plant species and total number of individuals from each collection included in ddRADseq study. Populations associated with the native nightshade species *Solanum umbelliferum* and *S. douglasii* are highlighted in yellow, samples from the solanaceous crop plants *Capsicum annuum* (pepper), *S. lycopersicum* (tomato) or *S. tuberosum* (potato) are in green, and samples from the native desert Solanaceae *Lycium andersonii* and *Physalis crassifolia* are highlighted in purple. While we often observed large numbers of potato psyllids on pepper and potato during our surveys, potato psyllids were exceedingly rare on tomatoes. Although we were able to find at least one potato psyllid via active sampling at each of the two tomato farms included in this study, this was only made possible via a great investment of time spent searching and, at one location (West Coast), we supplemented active collections with trapping via yellow sticky cards over the course of an entire week. Codes in parentheses following the total number of psyllids indicate the haplotype distribution (W = Western, C = Central, NW = Northwestern, SW = Southwestern).
**Table S2:** Sampling sites from which CLso‐positive psyllids were recovered. Host plant abbreviations correspond to *S. umbelliferum* (S. umb.), *Capsicum annuum* (C. ann.), *S. lycopersicum* (S. lys.).
**Table S3:** iNaturalist observations of *Bactericera cockerelli* collected from non‐solanaceous hosts throughout the Southwestern U.S.
**Table S4:** Mitochondrial COI sequencing results for 12 *B. cockerelli* collected from pine located on Mt. Whitney in the Sierra Nevada Mountains of California. The table also shows sequencing results for a portion of the *matK* barcoding gene amplified from samples of the pine tree from which psyllids were collected. Of the three species with *matK* sequences matching the sequence we amplified, only *Pinus contorta* (lodgepole pine) is known to occur in the Sierra Nevada mountains.
**Table S5:** Primer sets generated by Primer‐BLAST to amplify a portion of the *matK* gene from pine samples collected on Mt. Whitney.
**Table S6:** Psyllid samples from the RADseq analysis, which were also sequenced a second time using Illumina short‐read sequencing (whole genome). Sequences of *Candidatus* Carsonella ruddi were assembled from sequencing data for further analysis. Biosample accessions indicate the corresponding RADseq data deposited on NCBI Genbank.

## Data Availability

Data for this project are available on NCBI GenBank under BioProject PRJNA1433543 (ddRADseq data) and BioProject PRJNA1399511 (NGS‐Illumina data).
