# Supplementary material for: Ecological and Genomic Evidence of Cryptic Speciation in the Potato Psyllid (Bactericera cockerelli)
Source: Evol Appl. 2026 Oct 20;19(10):e70334. doi: 10.1111/eva.70334 (PMC13681000; doi:10.1111/eva.70334)
Supplement: Supplementary file 1 [file EVA-19-e70334-s001.docx]

Supplementary information for:

**Ecological and genomic evidence of cryptic speciation in the potato psyllid (*Bactericera cockerelli*)**

**Methods**

***Description of sampling habitats and seasonality***

We sampled psyllid populations as close in time as possible, but due to differences in phenology between host plant species and across climate zones, latitudinal gradients, and growing environments (irrigated vs. natural), psyllid sampling from various host plant species throughout diverse regions of California spanned a 12-month period. Much of the state of California has a mediterranean climate, characterized by hot, dry summers, and warm, wet winters. The native perennial nightshade *Solanum umbelliferum* thrives in this climate, dying back to bare stems for a period of dormancy throughout the summer, and re-growing shoots with the onset of late fall rains through winter and spring. In contrast, irrigated solanaceous crops in California are primarily planted in the spring and grow throughout the summer. Therefore, due to their divergent primary growing seasons, we sampled psyllids from *S. umbelliferum* populations in April-May 2022, and sampled psyllids from solanaceous crops in May-September 2022, with crop sampling occurring earlier in southern California and later in northern California, in accordance with latitudinal differences in planting and harvest dates. Although temporal separation between sampling the paired locations is not ideal, it reflects the reality of host availability in the landscape. While our design does not allow us to detect day-to-day movement of psyllid haplotypes between habitats, it still enables detection of haplotype movements among hosts that succeed each other in availability. For example, solanaceous crop planting occurs as *S. umbelliferum* is senescing in many of the locations we sampled (Table S1), thus, if psyllids present on *S. umbelliferum* are then moving to crops, our sampling scheme would detect this. Similarly, in the desert areas (Fig. 1) senescence of *Lycium* and *Physalis* hosts co-occurs with planting of solanaceous crops the desert valleys and our sampling scheme would also detect psyllid migration from the wild plants to the crop habitats that succeed them temporally, if such movement is occurring.

Weather and crop planting patterns are distinct in the inland desert region of southern California. Here, native Solanaceae, such as *Lycium andersonii* and *Physalis crassifolia* are typically dormant throughout the summer but may leaf out after late summer monsoon rains and continue growing through the fall and winter. Irrigation and relatively warm winters allow farmers to grow potatoes throughout the winter and early spring in this region. Thus, in addition to sampling potato psyllids from *S. umbelliferum* and solanaceous crops throughout more coastal and northern California in 2022 (Fig. S1), we sampled potato psyllids in the desert from *Lycium* and *Physalis* in February 2023 and from potatoes in March 2023 (Table S1).

***Sequence analysis***

We demultiplexed the RAD sequence data using the *process_radtags* function and default parameters in Stacks 2.6 (Catchen et al., 2011), merged paired-end reads and removed adaptor sequences using PEAR v0.9.11 [(Zhang *et al.*, 2014)](https://paperpile.com/c/VCrDUP/WSDh4), and used *BWA-mem2* v.2.2.1 [(Vasimuddin *et al.*, 2019)](https://paperpile.com/c/VCrDUP/ey3yP) to align reads to the *Bactericera cockerelli* reference genome generated by [(Kwak *et al.*, 2023)](https://paperpile.com/c/VCrDUP/BbmKm). Next, we used BCFtools (v1.19; (Li & Durbin, 2009)) mpileup to call SNPs and then filtered the genotypes for a minimum read depth of 8 (--minDP) and minor allele frequency of 5% (--maf) and excluded indels (--remove-indels) and sites with over 75% missing data (--max-missing) using VCFtools v0.1.16-18 (Danecek et al., 2011). We then used the missing-indv option in VCFtools to remove individuals missing genotype calls in more than 30% of retained loci from subsequent analysis. This left us with a total dataset of 111,587 SNPs across 590 individual psyllid samples.

***Analysis of genomic variation in the obligate symbionts of* B. cockerelli *haplotypes***

For a separate project exploring effector diversity in CLso variants, we had previously generated Illumina short-read sequencing data using DNA extracted from multiple CLso-infected individuals of three of the four psyllid mtCOI haplotypes (Western, Northwestern, and Southwestern) collected from sites included in this study (Bioproject: PRJNA1399511). Prior to library preparation, we performed whole genome amplification using the Repli-G kit from Qiagen. Libraries were prepared by staff at the UCR Genomics Core Facility using the NEB Ultra II FS kit (E7805L) and sequenced on an Illumina NextSeq platform to generate 300 bp paired-end reads, yielding an average of 13.8 GB raw data per sample. For a revision of the present manuscript, we mined this sequence data for reference-guided assembly of the genomes of the obligate endosymbiont *Candidatus* Carsonella ruddii for at least two samples of each psyllid haplotype (Table S6). We mapped quality filtered and trimmed reads from each psyllid sample to the *Candidatus* Carsonella ruddii reference genome (GCF_002009355.1) using BWA-MEM. We then sorted these alignments and indexed them with SAMtools, then calculated mapping statistics using *samtools* *flagstat* and *samtools* *coverage*. We identified SNPs across all mapped reads using *bcftools,* *mpileup* and *bcftools* *call*. Sample-specific *Carsonella* consensus genome sequences were generated using *bcftools* *consensus*. We aligned consensus genome sequences using MAFFT v7.505. We calculated pairwise genetic distances among *Carsonella* genomes from the aligned consensus sequences using the *ape* package in RStudio and visualized distances as a heatmap. To examine the genetic relationships among the *Carsonella* genomes across all psyllid samples, we performed principal component analysis (PCA) using high-quality biallelic SNPs. Variant data were converted to a genetic distance matrix using PLINK2 and used to calculate principal components, the first two of which were visualized in R using *ggplot2*.

**Figures**

**Figure S1.** Map of California showing 49 locations across 15 counties where potato psyllids were sampled from cultivated (pepper, potato, and tomato) and native (*Solanum umbelliferum, S. douglasii, Lycium andersonii,* and *Physalis crassifolia*) Solanaceae from spring 2022 to spring 2023.

**Figure S2.** Principal component analysis visualizing *Carsonella* SNP variation. PCA was performed using 4,326 high-quality SNPs identified across *Carsonella* genomes from 10 psyllid individuals (Table S6). Circles indicate individuals sampled from crop hosts and triangles indicate individuals sampled from wild hosts. The PCA for *Carsonella* SNPs mirrors the separation and clustering of psyllid haplotypes in the PCA generated from psyllid RADseq SNP data (Fig. 2) and the haplotype map (Fig. 4), providing an additional line of evidence showing clear lineage differentiation.

**Figure S3.** PCA plots of genetic variation between all potato psyllids of the [A] Northwestern haplotype (289 total individuals), [B] Western haplotype (247 total individuals), and [C] Southwestern haplotype (54 total individuals). Named sites indicate discrete clusters that separate from the main cluster containing individuals from multiple populations. For the Western haplotype, we did not detect distinct clusters of individuals from the UCR Ag Ops and UCI SCREC sites where putative inter-haplotype hybrids were detected. However, unlabeled points diverging from the main cluster contain many individuals from the early season collections in this site when putative hybrids were present.

**Figure S4.** Heatmap of Average Nucleotide Identities (ANI) between *Carsonella* genomes from individuals of different psyllid haplotypes. Higher ANI values (blues) indicate greater genomic similarity among *Carsonella* symbionts and lower ANI values (reds) indicate dissimilarity. NW = Northwestern, W = Western, and SW = Southwestern. Sample codes correspond to information in Table S6.

**Figure S5.** Maximum likelihood tree based on 3,917 nt alignment of seven concatenated MLST genes amplified from ‘*Ca*. Liberibacter solanacearum.’ 1,000 bootstraps (only values ≥70% shown here). Haplotypes are listed on the right. New sequences from this study are in bold text.

**Tables**

| **County** | **Location Name** | **Coordinates** | **Sampling Date(s)** | **Host Plant** | **Total # of psyllids (haplotype distribution)** |
| --- | --- | --- | --- | --- | --- |
|  |  |  |  |  |  |
| Fresno | TerraNova | 34°33'29.81"N 117°48'51.92"W | 2022-6-30 | *C. annuum* | 17 (all W) |
| Los Angeles | Allied | 34°41'22.55"N 118° 1'43.08"W | 2022-6-29 | *S. tuberosum* | 14 (all W) |
|  | Griffith | 34° 7'59.37"N 118°19'1.95"W | 2022-5-28 | *S. douglasii* | 2 (all NW) |
|  | Wild Walnut | 34° 8'1.40"N 118°37'57.35"W | 2022-4-17 | *S. umbelliferum* | 8 (all NW) |
|  | Thompson Creek | 34°7'44.67"N 117°43'28.23"W | 2022-4-20 | *S. umbelliferum* | 17 (all NW) |
| Monterey | San Lorenzo | 36°12'20.35"N 121°9'1.38"W | 2022-4-13 | *S. umbelliferum* | 16 (all NW) |
| Orange | UC Irvine SCREC | 33°41'20.84"N 117°43'21.93"W | 2022-5-12 | *S. tuberosum* | 17 (13 W, 3 NW, 1 SW) |
|  |  |  | 2022-09-9 | *C. annuum* | 12 (all W) |
|  | Santiago Oaks | 33°49'10.55"N 117°46'26.55"W | 2022-4-17 | *S. umbelliferum* | 3 (all NW) |
|  | Aliso Woods | 33°36'43.76"N 117°46'8.91"W | 2022-5-12 | *S. umbelliferum* | 7 (all NW) |
| Riverside | Prime Time | 33°31'35.00"N 115°59'16.84"W | 2022-5-20 | *C. annuum* | 1 (W) |
|  |  |  | 2022-5-27 | *C. annuum* | 4 (all W) |
|  | UCR AgOps | 33°57'53.89"N 117°20'26.73"W | 2022-5-04 | *S. tuberosum* | 16 (7 W, 3 C, 6 NW) |
|  |  |  | 2022-8-29 | *C. annuum* | 10 (all W) |
|  | Grant | 33°33'12.05"N 116°2'44.50"W | 2023-3-12 | *S. tuberosum* | 18 (all SW) |
|  | Joshua Tree | 33°48'3.70"N 115°46'55.04"W | 2023-2-11 | *L. andersonii* | 17 (all SW) |
|  |  | 33°43'5.01"N 115°48'32.85"W | 2023-2-11 | *P. crassifolia* | 5 (all SW) |
|  | Mecca Hills | 33°35'5.65"N 115°58'51.33"W | 2023-3-12 | *P. crassifolia* | 2 (all SW) |
|  | Sycamore Canyon | 33°56'58.50"N 117°19'7.75"W | 2022-4-19 | *S. umbelliferum* | 18 (all NW) |
|  | Motte | 33°48'16.88"N 117°15'18.04"W | 2022-4-19 | *S. umbelliferum* | 6 (all NW) |
|  | Cole | 33°33'50.38"N 117°15'13.22"W | 2022-4-22 | *S. umbelliferum* | 8 (all NW) |
|  | Emerson Oaks | 33°28'6.40"N 117°2'33.70"W | 2022-4-22 | *S. umbelliferum* | 18 (all NW) |
|  | Bogart | 33°58'49.85"N 116°57'7.34"W | 2022-4-22 | *S. umbelliferum* | 10 (all NW) |
|  | Shipley-Skinner | 33°36'49.27"N 117°1'9.95"W | 2022-5-13 | *S. umbelliferum* | 8 (all NW) |
|  | Corona | 33°50'40.86"N 117°36'20.71"W | 2022-4-20 | *S. umbelliferum* | 7 (all NW) |
| San Benito | Hollister | 36°51'3.54"N 121°18'36.52"W | 2022-7-08 | *C. annuum* | 14 (all W) |
|  |  |  | 2022-9-01 | *C. annuum* | 12 (all W) |
|  | San Juan Bautista (Pinnacle) | 36°51'33.35"N 121°30'10.99"W | 2022-9-01 | *S. tuberosum* | 7 (all W) |
| San Bernardino | Mojave | 35°12’07”N 115°45’46”W | 2023-5-05 | *L. andersonii* | 8 (all SW) |
| San Diego | West Coast | 33°15'22.66"N 117°14'37.23"W | 2022-6-10 | *S. lycopersicum* | 1 (W) |
|  |  |  | 2022-6-17 | *S. lycopersicum* | 3 (all W) |
|  | Anza-Borrego | 32°52'22.80"N 116°13'16.70"W | 2023-2-01 | *P. crassifolia* | 1 (SW) |
|  | Barrel Spring | 33°12'56.52"N 116°35'11.42"W | 2022-4-27 | *S. umbelliferum* | 17 (15 NW, 2 SW) |
|  | San Luis Rey | 33°15'21.08"N 116°47'44.74"W | 2022-4-27 | *S. umbelliferum* | 8 (all NW) |
|  | Elliot | 32°53'46.06"N 117°5'5.97"W | 2022-4-28 | *S. umbelliferum* | 4 (all NW) |
|  | Mission Trails | 32°49'23.05"N 117°4'40.95"W | 2022-4-28 | *S. umbelliferum* | 11 (all NW) |
|  | Dawson | 33°8'54.49"N 117°14'51.26"W | 2022-4-28 | *S. umbelliferum* | 6 (2 W, 4 NW) |
| San Joaquin | Morada | 38°3'52.85"N 121°21'55.00"W | 2022-7-06 | *C. annuum* | 1 (W) |
|  |  |  | 2022-8-31 | *C. annuum* | 15 (all W) |
|  | Zuckerman | 37°59'18.01"N 121°29'3.58"W | 2022-8-31 | *S. tuberosum* | 2 (all W) |
| San Luis Obispo | Talley | 35°10'12.79"N 120°31'43.63"W | 2022-6-30 | *C. annuum* | 4 (all W) |
|  |  |  | 2022-7-08 | *C. annuum* | 3 (all W) |
|  |  |  | 2022-9-01 | *C. annuum* | 15 (all W) |
| Santa Barbara | Cachuma Lake | 34°34'50.09"N 119°58'43.47"W | 2022-4-14 | *S. umbelliferum* | 1 (NW) |
|  | Carpinteria | 34°23'11.89"N 119°29'53.59"W | 2022-4-14 | *S. umbelliferum* | 18 (all NW) |
| Santa Clara | B&T | 36°59'47.72"N 121°31'19.74"W | 2022-7-08 | *C. annuum* | 14 (all W) |
|  |  |  | 2022-9-01 | *C. annuum* | 11 (all W) |
|  | Alum Rock | 37°23'50.31"N 121°47'56.59"W | 2022-4-13 | *S. umbelliferum* | 18 (all NW) |
| Sutter | Park | 39°4'37.56"N 121°52'44.68"W | 2022-7-07 | *C. annuum* | 1 (W) |
| Ventura | UC Hansen REC | 34°19'31.59"N 119°6'17.72"W | 2022-6-20 | *C. annuum* | 12 (all W) |
|  |  |  | 2022-9-14 | *C. annuum* | 8 (all W) |
|  | Deardorff | 34°11'1.50"N 119°2'40.50"W | 2022-6-20 | *C. annuum* | 17 (all W) |
|  |  |  | 2022-9-14 | *C. annuum* | 12 (all W) |
|  | CSU | 34°10'43.19"N 119°2'17.45"W | 2022-4-16 | *S. umbelliferum* | 17 (all NW) |
|  | Arroyo Verde | 34°17'8.79"N 119°13'33.63"W | 2022-4-16 | *S. umbelliferum* | 3 (all NW) |
|  | Dennison | 34°26'14.05"N 119°12'10.56"W | 2022-4-16 | *S. umbelliferum* | 8 (all NW) |
|  | Sulphur | 34°24'35.33"N 119°12'5.64"W | 2022-4-16 | *S. umbelliferum* | 8 (all NW) |
|  | Triunfo | 34°9'16.78"N 118°50'47.68"W | 2022-4-17 | *S. umbelliferum* | 14 (all NW) |
| Yolo | Full Belly | 38°51'34.23"N 122°12'48.45"W | 2022-7-07 | *C. annuum* | 1 (W) |
|  |  |  | 2022-9-01 | *C. annuum* | 16 (all W) |
|  | Rominger | 38°35'23.52"N 121°58'37.45"W | 2022-7-07 | *S. lycopersicum* | 1 (W) |
|  | Stebbins | 38°30'29.18"N 122°5'50.96"W | 2022-4-10 | *S. umbelliferum* | 17 (all NW) |

**Table S1:** Potato psyllid sampling locations, dates, host plant species, and total number of individuals from each collection included in ddRADseq study. Populations associated with the native nightshade species *Solanum umbelliferum* and *S. douglasii* are highlighted in yellow, samples from the solanaceous crop plants *Capsicum annuum* (pepper), *S. lycopersicum* (tomato), or *S. tuberosum* (potato) are in green, and samples from the native desert Solanaceae *Lycium andersonii* and *Physalis crassifolia* are highlighted in purple. While we often observed large numbers of potato psyllids on pepper and potato during our surveys, potato psyllids were exceedingly rare on tomatoes. Although we were able to find at least one potato psyllid via active sampling at each of the two tomato farms included in this study, this was only made possible via a great investment of time spent searching and, at one location (West Coast), we supplemented active collections with trapping via yellow sticky cards over the course of an entire week. Codes in parentheses following the total number of psyllids indicate the haplotype distribution (W = Western, C = Central, NW = Northwestern, SW = Southwestern).

| **Population code** | **Population name** | **Host plants** | **Total psyllids tested** | **Total CLso positive** | **mtCOI haplotype of CLso-positive psyllids** | **CLso haplotype(s)** |
| --- | --- | --- | --- | --- | --- | --- |
| AW | Aliso Woods | S. umb. | 7 | 1 | Northwestern | G |
| BS | Barrel Spring | S. umb. | 17 | 3 | Northwestern | B/Sumb2 |
| CB | Carpinteria Bluffs | S. umb. | 18 | 6 | Northwestern | B |
| CS | CSU Channel Islands | S. umb. | 17 | 4 | Northwestern | G |
| DF | Deardorff Farms | C. ann. | 29 | 6 | Western | A |
| EC | Elliot Chaparral Reserve | S. umb. | 4 | 2 | Northwestern | F |
| EO | Emerson Oaks Reserve | S. umb. | 18 | 2 | Northwestern | Sumb2 |
| SL | San Luis Rey turnout | S. umb. | 8 | 1 | Northwestern | Sumb2 |
| SC | Sycamore Canyon | S. umb. | 18 | 5 | Northwestern | B, B/Sumb2 |
| TC | Thompson Creek | S. umb. | 17 | 4 | Northwestern | G |
| TP | Triunfo Park | S. umb. | 14 | 3 | Northwestern | B |
| WC | West Coast Tomato | S. lys. | 4 | 1 | Western | B |
| WW | Wild Walnut Park | S. umb | 8 | 1 | Northwestern | B |

**Table S2.** Sampling sites from which CLso-positive psyllids were recovered. Host plant abbreviations correspond to *S. umbelliferum* (S. umb.), *Capsicum annuum* (C. ann.), *S. lycopersicum* (S. lys.).

| **Month, Day** | **Year** | **Reported host plant** | **Region** | **Number of psyllids** | **Link** |
| --- | --- | --- | --- | --- | --- |
| 03-28 | 2019 | Ponderosa pine | Mount Lemmon (Arizona) | at least two | <https://www.inaturalist.org/observations/21713667> |
| 04-03 | 2019 | Unspecified pine | Mount Lemmon (Arizona) | one | <https://www.inaturalist.org/observations/21966872> |
| 04-08 | 2019 | *Arctostaphylos* sp. | Mount Lemmon (Arizona) | many | <https://www.inaturalist.org/observations/22245331> |
| 04-15 | 2019 | Unspecified conifer | San Bernardino Mountains | one | <https://www.inaturalist.org/observations/22540776> |
| 04-30 | 2017 | Ceanothus papillosus | Santa Ana Mountains | one | <https://www.inaturalist.org/observations/9278986> |
| 05-09 | 2025 | *Arctostaphylos* sp. | San Bernardino Mountains | at least nine | <https://www.inaturalist.org/observations/280320009> |
| 05-14 | 2023 | Appears to be mistletoe | Santa Rosa/San Jacinto Mountains | one | <https://www.inaturalist.org/observations/166523610> |
| 05-14 | 2024 | Appears to be pine | Grand Canyon National Park (Arizona) | At least one | <https://www.inaturalist.org/observations/218463258> |
| 06-11 | 2017 | Artemisia tridentata | San Bernardino Mountains | at least two | <https://www.inaturalist.org/observations/9261522> |
| 06-25 | 2023 | Bristlecone pine | Panamint Mountains | many | <https://www.inaturalist.org/observations/169888223> |
| 07-26 | 2024 | *Pinus balfouriana*  (this manuscript) | Sierra Nevada Mountains | many | <https://www.inaturalist.org/observations/232155025> |
| 07-31 | 2022 | *Arctostaphylos* sp. | San Gabriel Mountains | many | <https://www.inaturalist.org/observations/129056333> |
| 08-31 | 2024 | *Ericameria nauseosa* (rabbitbrush) | San Bernardino Mountains | at least five | <https://www.inaturalist.org/observations/239712898> |
| 09-11 | 2016 | *Ericameria* sp. (rabbitbrush) | Sierra Nevada Mountains | many | <https://www.inaturalist.org/observations/4104884> |
| 09-24 | 2016 | *Arctostaphylos pringlei* | Santa Rosa/San Jacinto Mountains | one | <https://www.inaturalist.org/observations/4219612> |
| 09-26 | 2024 | *Garrya flavescens* (silktassel) | Santa Rosa/San Jacinto Mountains | at least two | <https://www.inaturalist.org/observations/286174297> |
| 10-22 | 2024 | *Ericameria* sp. (rabbitbrush) | Red Rock Canyon State Park (Nevada) | many | <https://www.inaturalist.org/observations/248840643> |
| 11-12 | 2020 | *Arctostaphylos* sp. | San Bernardino Mountains | one | <https://www.inaturalist.org/observations/64757165> |

**Table S3.** iNaturalist observations of *Bactericera cockerelli* collected from non-solanaceous hosts throughout the Southwestern U.S.

| **Sample type** | **SampleNo.** | **Consensus length (nt)** | **Top BLAST hit**  **(NCBI accession)** | **Haplotype or species** | **Query coverage** | **Percent Identity** |
| --- | --- | --- | --- | --- | --- | --- |
| Psyllid | 1 | 460 | KC305359.1 | Southwestern | 100 | 99.78 |
| Psyllid | 2 | 405 | KC305359.1 | Southwestern | 100 | 100 |
| Psyllid | 3 | 460 | KC305359.1 | Southwestern | 100 | 100 |
| Psyllid | 4 | 460 | KC305359.1 | Southwestern | 100 | 100 |
| Psyllid | 5 | 460 | KC305359.1 | Southwestern | 100 | 100 |
| Psyllid | 6 | 460 | KC305359.1 | Southwestern | 100 | 99.57 |
| Psyllid | 7 | 460 | KC305359.1 | Southwestern | 100 | 100 |
| Psyllid | 8 | 460 | KC305359.1 | Southwestern | 100 | 100 |
| Psyllid | 9 | 460 | KC305359.1 | Southwestern | 100 | 99.57 |
| Psyllid | 10 | 411 | KC305359.1 | Southwestern | 100 | 100 |
| Psyllid | 11 | 413 | KC305359.1 | Southwestern | 100 | 99.27 |
| Psyllid | 12 | 397 | KC305359.1 | Southwestern | 100 | 99.75 |
| Pine | n/a | 529 | MH612863.1 | *Pinus contorta* | 100 | 100 |
|  |  |  | FJ580113.1 | *Pinus virginiana* | 100 | 100 |
|  |  |  | JN854217.1 | *Pinus clausa* | 100 | 100 |

**Table S4.** Mitochondrial COI sequencing results for 12 *B. cockerelli* collected from pine located on Mt. Whitney in the Sierra Nevada Mountains of California. The table also shows sequencing results for a portion of the *matK* barcoding gene amplified from samples of the pine tree from which psyllids were collected. Of the three species with *matK* sequences matching the sequence we amplified, only *Pinus contorta* (lodgepole pine) is known to occur in the Sierra Nevada mountains.

| Primer Name | Primer Sequence (5’-3’) | G/C Content | Tm (℃) | Primer Locus (relative to gene sequence) |
| --- | --- | --- | --- | --- |
| matk2F | CCGAACCGTATAGGGTCTGTT | 52.4% | 63.2 | 815-835 |
| matk2R | GCCAAATTCGTTCTCTCTGCG | 52.4% | 63.6 | 1345-1325 |

**Table S5.** Primer sets generated by Primer-BLAST to amplify a portion of the *matK* gene from pine samples collected on Mt. Whitney.

| **RAD-seq BioSample ID** | **Sample IDs** | **Haplotype** | **Collection Site (see Table S1)** |
| --- | --- | --- | --- |
| SAMN55909173 | CSU9-NW | Northwestern | CSU Channel Islands Creek |
| SAMN55909170 | CSU14-NW | Northwestern | CSU Channel Islands Creek |
| SAMN55909182 | EOR13a-NW | Northwestern | Emerson Oaks Reserve |
| SAMN55909183 | EOR18-NW | Northwestern | Emerson Oaks Reserve |
| SAMN55909179 | DFS3-W | Western | Deardorff Farms, Cawelti Field (September) |
| SAMN55909177 | DFS10-W | Western | Deardorff Farms, Cawelti Field (September) |
| SAMN55909199 | JTL7-SW | Southwestern | Joshua Tree National Park, Smoketree Wash (*Lycium*) |
| SAMN55909200 | JTL8-SW | Southwestern | Joshua Tree National Park, Smoketree Wash (*Lycium*) |
| SAMN55909180 | ELL2-NW | Northwestern | Elliot Chaparral Reserve |
| SAMN55909181 | ELL4-NW | Northwestern | Elliott Chaparral Reserve |

**Table S6.** Psyllid samples from the RADseq analysis, which were also sequenced a second time using Illumina short-read sequencing (whole genome). Sequences of *Candidatus* Carsonella ruddi were assembled from sequencing data for further analysis. Biosample accessions indicate the corresponding RADseq data deposited on NCBI Genbank.

**References**

Catchen, J. M., A. Amores, P. Hohenlohe, W. Cresko, and J. H. Postlethwait. 2011. “Stacks: Building and Genotyping Loci de Novo From Short‐Read Sequences.” *G3: Genes, Genomes, Genetics* 1: 171–182.

Danecek, P., A. Auton, G. Abecasis, et al. 2011. “The Variant Call Format and VCFtools.” *Bioinformatics* 27: 2156–2158.

Kwak, Y., J. A. Argandona, P. H. Degnan, and A. K. Hansen. 2023. “Chromosomal‐Level Assembly of *Bactericera cockerelli* Reveals Rampant Gene Family Expansions Impacting Genome Structure, Function and Insect‐Microbe‐Plant‐Interactions.” *Molecular Ecology Resources* 23: 233–252.

Li, H., and R. Durbin. 2009. “Fast and Accurate Short Read Alignment With Burrows‐Wheeler Transform.” *Bioinformatics* 25: 1754–1760.

Vasimuddin, M., S. Misra, H. Li, and S. Aluru. 2019. “Efficient Architecture‐Aware Acceleration of BWA‐MEM for Multicore Systems.” In *2019 IEEE International Parallel and Distributed Processing Symposium (IPDPS)*, 314–324. IEEE.

Zhang, J., K. Kobert, T. Flouri, and A. Stamatakis. 2014. “PEAR: A Fast and Accurate Illumina Paired‐End reAd mergeR.” *Bioinformatics* 30: 614–620.
